# Genetic Engineering as a Strategy to Improve the Therapeutic Efficacy of Mesenchymal Stem/Stromal Cells in Regenerative Medicine

**DOI:** 10.3389/fcell.2020.00737

**Published:** 2020-08-21

**Authors:** Patricia Kauanna Fonseca Damasceno, Thaís Alves de Santana, Girlaine Café Santos, Iasmim Diniz Orge, Daniela Nascimento Silva, Juliana Fonseca Albuquerque, Giulia Golinelli, Giulia Grisendi, Massimo Pinelli, Ricardo Ribeiro dos Santos, Massimo Dominici, Milena Botelho Pereira Soares

**Affiliations:** ^1^Gonçalo Moniz Institute, Oswaldo Cruz Foundation (FIOCRUZ), Salvador, Brazil; ^2^Health Institute of Technology, SENAI CIMATEC, Salvador, Brazil; ^3^Division of Oncology, Laboratory of Cellular Therapy, University of Modena and Reggio Emilia, Modena, Italy; ^4^Division of Plastic Surgery, Department of Medical and Surgical Sciences for Children & Adults, University of Modena and Reggio Emilia, Modena, Italy; ^5^National Institute of Science and Technology for Regenerative Medicine (INCT-REGENERA), Rio de Janeiro, Brazil

**Keywords:** mesenchymal stem/stromal cells, genetic engineering, regenerative medicine, cell therapy, gene therapy

## Abstract

Mesenchymal stem/stromal cells (MSCs) have been widely studied in the field of regenerative medicine for applications in the treatment of several disease settings. The therapeutic potential of MSCs has been evaluated in studies *in vitro* and *in vivo*, especially based on their anti-inflammatory and pro-regenerative action, through the secretion of soluble mediators. In many cases, however, insufficient engraftment and limited beneficial effects of MSCs indicate the need of approaches to enhance their survival, migration and therapeutic potential. Genetic engineering emerges as a means to induce the expression of different proteins and soluble factors with a wide range of applications, such as growth factors, cytokines, chemokines, transcription factors, enzymes and microRNAs. Distinct strategies have been applied to induce genetic modifications with the goal to enhance the potential of MCSs. This review aims to contribute to the update of the different genetically engineered tools employed for MSCs modification, as well as the factors investigated in different fields in which genetically engineered MSCs have been tested.

## Introduction

In the field of stem cell therapy, mesenchymal stem/stromal cells (MSCs) have been widely used in a large number of *in vitro* and *in vivo* studies, as well as in approximately 1000 clinical trials. These are multipotent stem cells that must meet minimum criteria, such as plastic adherence, expression of specific surface markers and the ability to differentiate in adipocytes, chondrocytes and osteocytes (Dominici et al., 2006). Compared to other stem cells, such as embryonic and induced pluripotent stem cells, the use of MSCs has the advantage of being considered safer, regarding the possibility of tumor formation. Additionally, these cells are easier to obtain from different autologous or allogeneic sources (Kim and Park, 2017). Moreover, MSCs have the capacity to secrete a repertoire of factors with immunomodulatory and anti-apoptotic potential, as well as factors associated to angiogenesis and tissue regeneration (Murphy et al., 2013).

While in numerous studies the therapeutic effects of MSCs have been shown, in others the transplantation of MSCs did not lead to the desired effect, as evaluated in different disease models (Meyer et al., 2006; Huang B. et al., 2012; Sajic et al., 2012). Some studies have shown that transplanted MSCs presented a poor survival rate and proliferation (Shi and Li, 2008; Park J.S. et al., 2015; Li et al., 2016; Silva et al., 2018c; Zhao et al., 2019), possibly due to the hostile microenvironment of lesioned tissues, which could lead to nutrient deprivation and cell death (Moya et al., 2018). Moreover, partial beneficial effects may be enhanced by expression of specific factors capable of promoting desired effects in the target disease setting. Therefore, approaches aiming to enhance the efficacy of MSC transplantation are needed in order to achieve the suitable results.

Genetic engineering of MSCs has been studied in the past years with the purpose of enhancing the therapeutic potential of these cells and improving the outcomes after transplantation. This has been achieved using non-viral or viral vectors to induce the expression of different factors, depending on the desired results, such as increasing their survival and proliferation rate and improving their pro-regenerative capacity (Sage et al., 2016; Foppiani et al., 2019). In this review, we discuss the current methods employed in the generation of genetically modified MSCs and the results obtained with the expression of different factors and the main disease settings in which this modality of cell and gene therapy has been investigated. Table 1 summarizes the modification agents, cell source, genetic engineering method and applications of diverse studies described in this review.

**TABLE 1:** Genetic modifications in MSCs and disease models tested.

Factor overexpressed	MSC source	Method	Disease	References
Akt	Mouse bone marrow	Retrovirus	Myocardial infarction	Noiseux et al., 2006
	Rabbit amniotic fluid	Lentivirus	Heart ischemia-reperfusion injury	Wang et al., 2016
	Human umbilical cord	Adenovirus	Acute myocardial infarction	Ma et al., 2017
	Rat bone marrow	Retrovirus	Myocardial infarction	Gnecchi et al., 2006, 2009
Akt and angiopoietin-1 (Ang-1)	Rat bone marrow	Adenovirus	Infarcted heart	Jiang et al., 2006
Akt and Wnt11	Rat bone marrow	AAV	Hypoxia/reoxygenation-induced cardiomyocyte apoptosis	Chen B. et al., 2018
Angiogenin	Yorkshire pig bone marrow	Adenovirus	Myocardial chronic ischemic	Huang et al., 2006
Angiopoietin-1 (Ang1)	Mouse bone marrow	Plasmid transfection	Acute respiratory distress syndrome	Mei et al., 2007
	Rat bone marrow	Lentivirus	Phosgene-induced acute lung injury	Shao et al., 2018
Angiotensin II type 2 receptor	Human bone marrow	Lentivirus	LPS-induced acute lung injury	Xu et al., 2018
Angiotensin-converting enzyme 2 (ACE2)	Mouse bone marrow	Lentivirus	Acute lung injury	He et al., 2015
Arginine decarboxylase	Human adipose tissue	Retrovirus	Spinal cord injury	Park Y.M. et al., 2015
ATP7B	Human bone marrow	Retrovirus	Wilson’s disease	Sauer et al., 2010
Basic fibroblast growth factor (bFGF)	Human bone marrow	Lentivirus	Angiogenesis	Fierro et al., 2011
	Rat bone marrow	Lentivirus	Ischemic disease	Zhang J.C. et al., 2014
B-cell lymphoma protein 2 (BCL-2)	Rat bone marrow	AAV	Liver cirrhosis	Jin et al., 2016
Brain-derived neurotrophic factor (BDNF)	Human umbilical cord blood	Plasmid transfection	Neurological injury and disease	Lim et al., 2011
	Human bone marrow	Lentivirus	Neuronal degeneration	Scheper et al., 2019
	Rat bone marrow	Adenovirus	Spinal cord injury	Zhao et al., 2013
	Rat bone marrow	Adenovirus	Neonatal stroke	Van Velthoven et al., 2013
	Rat bone marrow	Lentivirus	Ischemia	Zhou et al., 2017
C1q/tumor necrosis factor-related protein-3 (CTRP3)	Mouse bone marrow	Lentivirus	Myocardial infarction	Zhang Z. et al., 2019
C-C chemokine receptor type 2 (CCR2)	Human bone marrow	Lentivirus	Ischemic stroke	Zhang Y. et al., 2018
C-C chemokine receptor type 1 CCR1	Mouse bone marrow	Retrovirus	Injured myocardium	Huang et al., 2010
Cellular repressor of E1A-stimulated genes (CREG)	Rat bone marrow	Adenovirus	Myocardial infarction	Deng et al., 2010
Cerebral dopamine neurotrophic factor (CDNF)	Rat bone marrow	Lentivirus	Spinal cord injury	Zhao et al., 2014
Ciliary neurotrophic factor (CNTF)	Rat bone marrow	Plasmid transfection	Traumatic injury to the central nervous system	Abbaszadeh et al., 2015
c-MYC	Mouse bone marrow	Retrovirus	Osteosarcoma	Shimizu et al., 2010
Csx/Nkx2.5 and GATA4	Mouse bone marrow	Retrovirus	Stochastic cardiomyogenic fate	Yamada et al., 2007
	Mouse bone marrow	Retrovirus	Myocardial infarction	Gao et al., 2011
CXC chemokine receptor 4 (CXCR4)	Human umbilical cord	Lentivirus	Radiation- induced lung injury	Zhang C. et al., 2019
	Mouse bone marrow	Retrovirus	Breast cancer	Kalimuthu et al., 2017
	Mouse bone marrow	Lentivirus	Inflammatory bowel and cancer	Zheng et al., 2019
	Rat bone marrow	Retrovirus	Myocardial infarction	Cheng et al., 2008
	Rat bone marrow	Adenovirus	Ischemic heart injury	Wu S. Z. et al., 2017
	Rat bone marrow	Lentivirus	Myocardial infarction	Kang et al., 2015
	Mouse bone marrow	Lentivirus	Acute kidney injury	Liu et al., 2013
	Rat bone marrow	Adenovirus	Liver regeneration	Du et al., 2013
	Rat bone marrow	Lentivirus	Myocardial neovascularization	Liang et al., 2012
	Mouse bone marrow	Adenovirus	Myocardial infarction	Huang Z. Y. et al., 2012
	Rat bone marrow	Adenovirus	Myocardial infarction	Zhang et al., 2008
CXC chemokine receptor 7 (CXCR7)	Rat bone marrow	Lentivirus	Acute lung injury	Shao et al., 2019
Cytosine deaminase (CD) and Herpes simplex virus thymidine kinase (HSV-tk)	Human umbilical cord blood	Lentivirus	Ovarian cancer	Jiang et al., 2014
Drosophila mothers against decapentaplegic 7 (Smad7)	Rat bone marrow	Lentivirus	Hepatic fibrosis	Wu S. P. et al., 2017
Endothelial nitric oxide synthase (eNOS)	Rat bone marrow	Adenovirus	Endothelial dysfunction	Kanki-Horimoto et al., 2006
	Mouse bone marrow	Adenovirus	Myocardial Infarction	Chen L. et al., 2017
Ephrin-B2	Human bone marrow	Plasmid transfection	Ischemic tissues	Duffy et al., 2010
Erythropoietin (EPO)	Mouse bone marrow	Retrovirus	Myocardial infarction	Copland et al., 2008
	Mouse bone marrow	Retrovirus	Cancer immunotherapy	Campeau et al., 2009
Extracellular regulating kinase 1/2 (ERK1/2)	Rat bone marrow	Lentivirus	Ischemic stroke	Gao et al., 2019
Fibroblast growth factor 21 (FGF21)	Mouse bone marrow	Lentivirus	Traumatic brain injury	Shahror et al., 2019
Fibroblast growth factor 4 (FGF4)	Rat bone marrow	Lentivirus	Liver cirrhosis	Wang et al., 2015
Follistatin-like 1 (Fstl1)	Mouse bone marrow	Lentivirus	Myocardial infarction	Shen et al., 2019
Forkhead box protein (Foxa2)	Human adipose tissue	Plasmid transfection	Acute liver injury	Chae et al., 2019
	Rat bone marrow	Plasmid transfection	Hepatic diseases	Cho J.W. et al., 2012
GATA-4	Mouse bone marrow	Lentivirus	Myocardial infarction	He et al., 2019
	Rat bone marrow	Retrovirus	Myocardial ischemia	Yu B. et al., 2016
	Rat bone marrow	Retrovirus	Heart ischemic injury	Li et al., 2014
Glial-derived neurotrophic factor (GDNF)	Human adipose	Lentivirus	Renal interstitial fibrosis	Wang Z. et al., 2019
	Rat bone marrow	Plasmid transfection	Neurodegenerative diseases	Noori-Zadeh et al., 2014
	Human bone marrow	Adenovirus	Nephrotoxic serum nephritis (NSN)	Huang Z. Y. et al., 2012
	Rat bone marrow	Adenovirus	Intracerebral hemorrhage (ICH)	Yang et al., 2011
Glucocorticoid-induced tumor necrosis factor–related receptor (GITR)	Human bone marrow	Plasmid transfection	Small cell lung cancer	Kopru et al., 2018
Glycogen synthase kinase-3β (GSK-3β)	Mouse bone marrow	Adenovirus	Myocardial infarction	Cho et al., 2011
Granulocyte chemotactic protein (GCP)-2	Human adipose tissue	Lentivirus	Myocardial infarction	Kim et al., 2012
Granulocyte-colony stimulating factor (G-CSF)	Mouse bone marrow	Lentivirus	Chagas disease cardiomyopathy	Silva et al., 2018b
Heme oxygenase-1 (HO-1)	Mouse adipose tissue	Plasmid transfection	Heart ischemic injury	Preda et al., 2014
	Rat bone marrow	Lentivirus	Acute lung injury	Chen X. et al., 2018
	Dog adipose tissue	Lentivirus	Spinal cord injury	Lee et al., 2017
	Human embryonic stem cell	Lentivirus	Myocardial infarction	Kearns-Jonker et al., 2012
	Rabbit adipose tissue	Adenovirus	Myocardial infarction	Yang et al., 2012
Hepatocyte growth factor (HGF)	Human bone marrow	Retrovirus	Bladder outlet obstruction	Song et al., 2012
	Rat bone marrow	Adenovirus	Hepatocirrhosis	Zhang Y. et al., 2018
	Human umbilical cord	Lentivirus	Myocardial infarction	Zhao et al., 2016
	Human bone marrow	Adenovirus	Liver fibrosis	Lai et al., 2015
	Human umbilical cord	Adenovirus	Injured sinonasal mucosa	Li et al., 2015
	Rat bone marrow	Lentivirus	Liver damage caused by radiotherapy	Zhang J. et al., 2014
	Human umbilical cord	Adenovirus	Parkinson’s disease	Liu et al., 2014
	Human umbilical cord blood	Plasmid transfection	Liver fibrosis	Seo et al., 2014
	Rat bone marrow	Adenovirus	Pulmonary arterial hypertension	Guo et al., 2013
	Human bone marrow	Lentivirus	Spinal cord injury	Jeong et al., 2012
	Rat bone marrow	Adenovirus	Post-ischemic heart failure	Guo et al., 2008
	Pig adipose tissue	Lentivirus	Intramyocardial transplant	Gómez-Mauricio et al., 2016
Hepatocyte nuclear factor 4α (HNF4α)	Human umbilical cord	Lentivirus	Hepatocellular carcinogenesis (HCC)	Wu et al., 2016
	Mouse bone marrow	Adenovirus	Liver cirrhosis	Ye et al., 2019
Human a1-antitrypsin (hAAT)	Mouse adipose tissue	Lentivirus	Disorders of liver metabolism	Di Rocco et al., 2012
Human elastin	Rat bone marrow	Adenovirus	Myocardial infarction	Li et al., 2012
Human N-cadherin	Human umbilical cord blood	Lentivirus	Myocardial infarction	Lee et al., 2012
Hypoxia inducible factor (HIF)-1α.	Human bone marrow	Lentivirus	Angiogenesis	Razban et al., 2012
	Rat bone marrow	Adenovirus	Ischemic cerebrovascular disease	Yang et al., 2014
	Sheep bone marrow	Plasmid transfection	Acute myocardial infarction	Hnatiuk et al., 2016
IL-1Ra	Rats amniotic fluid	Lentivirus	Fulminant hepatic failure	Zheng et al., 2012
IL-33	Rat bone marrow	Lentivirus	Myocardial infarction	Chen Y. et al., 2017
IL-4	Human adipose tissue	Lentivirus	Multiple sclerosis	Payne et al., 2012
Insulin growth factor like-I (IGF-I)	Rat bone marrow	Adenovirus	Myocardial infarction	Haider et al., 2008
	Mouse bone marrow	Retrovirus	Renal failure-induced anemia	Kucic et al., 2008
	Pig adipose tissue	Lentivirus	Intramyocardial transplant	Gómez-Mauricio et al., 2016
	Mouse bone marrow	Retrovirus	Spinal cord injury	Allahdadi et al., 2019
	Mouse bone marrow	Lentivirus Adenovirus	Chagas disease cardiomyopathy	Silva et al., 2018a
	Mouse bone marrow	Adenovirus	Cirrhosis	Fiore et al., 2015
Integrin linked kinase (ILK)	Mini-pig bone marrow	Adenovirus	Myocardial infarction	Mao Q. et al., 2014
	Rats bone marrow	Adenovirus	Myocardial infarction	Mao et al., 2013
Integrin α4	Rat bone marrow	Lentivirus	Cerebral Embolism	Cui et al., 2017
Intercellular Adhesion Molecule 1 (ICAM-1)	Mouse compact bone	Plasmid transfection	Inflammatory bowel disease	Li et al., 2019
Interferon-beta (IFN-β)	Dog adipose tissue	Lentivirus	Melanoma	Han et al., 2015
	Mouse bone marrow	Lentivirus	Breast cancer	Ling et al., 2010
Interleukin-10	Mouse bone marrow	CRISPRa system	Myocardial infarction	Meng et al., 2019
	Rat bone marrow	Lentivirus	Traumatic brain injury	Maiti et al., 2019
	Human amniotic fluid	Adenovirus	Liver fibrosis	Choi et al., 2019
	Mouse bone marrow	Retrovirus	Acute lung injury	Wang et al., 2018
	Human bone marrow	AAV	Acute Ischemic Stroke	Nakajima et al., 2017
	Rat bone marrow	Adenovirus	Myocardial infarction	Meng et al., 2017
Klotho	Mouse bone marrow	Adenovirus	Acute kidney injury	Zhang F. et al., 2018
Large Tumor Suppressor gene 2 (LATS2)	Mouse bone marrow	Lentivirus	Acute respiratory distress syndrome	Dong and Li, 2019
Leptin	Human bone marrow	Lentivirus	Myocardial infarction	Yang et al., 2018
LIM-homeobox transcription factor islet-1 (ISL1)	Human bone marrow	Lentivirus	Myocardial infarction	Xiang et al., 2018
Lipocalin2 (Lcn2)	Rats bone marrow	Plasmid transfection	Kidney injury	Halabian et al., 2014
Liver X receptor-α (LXRα)	Mouse bone marrow	Retrovirus	Pathophysiology of the renin-angiotensin system	Matsushita et al., 2010
Mammalian achaete-scute homologue-1 (Mash1)	Rat bone marrow	Lentivirus	Neurodegenerative disorders	Wang K. et al., 2013
MCT4	Mouse bone marrow	Retrovirus	Myocardial ischemia	Saraswati et al., 2015
miR-1	Mouse bone marrow	Lentivirus	Myocardial infarction	Huang et al., 2013b
miR-101-3p	Human bone marrow	Lentivirus	Oral cancer	Xie et al., 2019
miR-124	Rat bone marrow	Lentivirus	Spinal cord injury	Zou et al., 2014
miR-126	Mouse bone marrow	Lentivirus	Ischemic angiogenesis	Huang et al., 2013a
miR-133	Rat bone marrow	Lentivirus	Acute myocardial infarction	Chen L. et al., 2017
miR-133b	Rat bone marrow	Lentivirus	Stroke	Xin et al., 2017
miR-16−5p	Human bone marrow	Plasmid transfection	Colorectal cancer	Xu et al., 2019
miR-199a	Human bone marrow	Plasmid transfection	Glioma	Yu et al., 2019
miR-199a-3p	Human bone marrow	miRNA transfection	Renal ischemia/reperfusion injury	Zhu et al., 2019
miR-211	Rat and human bone marrows	Lentivirus	Adverse post-MI remodeling	Hu et al., 2016
miR-23a	Rat bone marrow	Lentivirus	Myocardial infarction	Mao J. et al., 2014
miR-30b-3p	Mouse bone marrow	Lentivirus	Acute lung injury	Yi et al., 2019
miR-34a	Human bone marrow	Lentivirus	Glioblastoma	Wang B. et al., 2019
miR-705	Mouse bone marrow	Lentivirus	Ischemic brain injury	Ji et al., 2017
miR-let-7d or miR-154	Human bone marrow	Lentivirus	Lung injury	Huleihel et al., 2017
miRNA-181	Human umbilical cord blood	Lentivirus	Myocardial ischemia-reperfusion injury	Wei et al., 2019
miRNA-21	Rat bone marrow	Lentivirus	Intracerebral hemorrhage	Zhang H. et al., 2018
miRNA-21	Rat bone marrow	Lentivirus	Myocardial damage	Zeng et al., 2017
miRNA-25	Rat bone marrow	Lentivirus	Transient spinal cord ischemia	Zhao et al., 2018
as-miR-937	Mouse bone marrow		Alzheimer’s Disease	Liu et al., 2015
Monocyte chemoattractant protein-induced protein 1 (MCPIP1)	Mouse bone marrow	Retrovirus	Myocardial repair	Labedz-Maslowska et al., 2015
Neuregulin 1 (NRG1)	Human adipose tissue	Adenovirus	Cerebral ischemia	Ryu et al., 2019
Neuregulin 4 (Nrg4)	Mouse adipose tissue	Lentivirus	Insulin resistance and hepatic steatosis	Wang W. et al., 2019
Neurotrophin-3 (NT-3)	Rat bone marrow	Plasmid transfection	Parkinson disease	Moradian et al., 2017
Nuclear factor (erythroid-derived 2)-like 2 (Nrf2)	Human amniotic fluid	Lentivirus	Acute lung injury	Zhang et al., 2017
*Oct4* and *Sox2*	Human adipose tissue	Plasmid transfection	Liver injury	Han et al., 2014
p130 and E2F4	Mouse bone marrow	Lentivirus	Acute respiratory distress syndrome	Zhang X. et al., 2019
PARKIN	Human Wharton’s jelly	Plasmid transfection	Parkinson’s disease	Bonilla-Porras et al., 2018
Periostin	Rat bone marrow	Lentivirus	Myocardial infarction	Cho Y.H. et al., 2012
Pigment epithelial-derived factor (PEDF)	Human bone marrow	Lentivirus	Hepatocellular carcinoma	Gao et al., 2010
	Human or rat bone marrow	Plasmid transfection	Cerebral ischemia-reperfusion injury	Huang Y. et al., 2018
	Mouse bone marrow	AAV	Glioma	Wang Q. et al., 2013
Receptor activity-modifying protein 1 (RAMP1)	Rabbit bone marrow	Adenovirus	Carotid angioplasty and myocardial infarction	Shi et al., 2014
Receptor tyrosine kinase-like orphan receptor 2 (ROR2)	Mouse bone marrow	Lentivirus	Acute respiratory distress syndrome (ARDS)	Cai et al., 2016
Sirtuin 1 (Sirt 1)	Mouse bone marrow	Adenovirus	Prostate cancer	Yu Y. et al., 2016
Sonic Hedgehog (Shh)	Mouse bone marrow	Lentivirus	Inflamed stomach	Donnelly et al., 2014
	Rat bone marrow	Lentivirus	Spinal cord injury	Jia et al., 2014
SRC3-specific short hairpin RNA (sh-SRC3)	Human bone marrow	Lentivirus	Multiple myeloma	Ji et al., 2017
sST2	Human adipose tissue	Lentivirus	Occupational asthma	Martínez-González et al., 2014
	Human adipose tissue	Lentivirus	Endotoxin-induced acute lung injury	Martínez-González et al., 2013
Stromal cell derived factor-1 (SDF-1)	Rat bone marrow	Lentivirus	Acute myocardial infarction	Unzek et al., 2007
	Mouse bone marrow	Lentivirus	Post-acute myocardial infarction	Mayorga et al., 2017
	Human umbilical cord blood	Plasmid transfection	Myocardial infarction	Gong et al., 2019
	Rat and human bone marrows	Retrovirus	Spinal cord injuries	Stewart et al., 2017
Telomerase (TERT) and myocardin (MYOCD)	Mouse adipose tissue	Lentivirus	Ischemic cardiovascular diseases	Madonna et al., 2019
Thioredoxin-1 (Trx-1)	Human umbilical cord	Adenovirus	Acute radiation injury (ARI)	Hu et al., 2013
Tissue matrix metalloproteinase inhibitor 2 (TIMP2)	Human umbilical cord	Lentivirus	Myocardial infarction	Ni et al., 2019
TNF-related apoptosis-inducing ligand (TRAIL)	Human bone marrow	Lentivirus	Non-small cell lung cancer	Xia et al., 2013
	Human adipose tissue	Plasmid transfection	Glioblastoma multiforme	Jiang et al., 2016
	Human adipose tissue	Lentivirus	Pancreatic ductal adenocarcinoma	Spano et al., 2019
	Human bone marrow	Lentivirus	Colorectal carcinoma	Mueller et al., 2011
	Human bone marrow	Lentivirus	Metastatic lung tumors	Loebinger et al., 2009
	Human bone marrow	Lentivirus	Cancer cell lines	Yuan et al., 2017
Transforming growth factor (TGF)β1	Human bone marrow	Lentivirus	Angiogenesis	Fierro et al., 2011
	Rat bone marrow	Lentivirus	Renal ischemia/reperfusion injury	Cai et al., 2019
Tropomyosin receptor kinase A (TrkA)	Rat bone marrow	Lentivirus	Peripheral nerve injury	Zheng et al., 2017
Tumor necrosis factor α (TNF α)	Human adipose tissue	Retrovirus	Lung metastases	Tyciakova et al., 2017
Tyrosine kinase C (TrkC)	Rat bone marrow	Adenovirus	Spinal cord injury	Ding et al., 2013
	Rat bone marrow	Adenovirus	Demyelinated spinal cord	Ding et al., 2015
Urokinase plasminogen activator	Human umbilical cord blood	Adenovirus	Tumor tropism	Pulukuri et al., 2010
Vascular endothelial growth factor (VEGF)	Human bone marrow	Lentivirus	Peripheral nerve injury	Man et al., 2016
	Sheep bone marrow	Plasmid transfection	Myocardial infarction	Locatelli et al., 2015
	Mouse bone marrow	Plasmid transfection	Alzheimer’s disease	Garcia et al., 2014
	Rat bone marrow	Lentivirus	Cardiac arrest	Zhou et al., 2017
	Mouse bone marrow	Adenovirus	Heart infarction	Wang et al., 2006
Wnt11	Rat bone marrow	Retrovirus	Cardiac ischemic Injury	Zuo et al., 2012
β-catenin	Mouse bone marrow	Lentivirus	Acute respiratory distress syndrome (ARDS)	Cai et al., 2015

## MSC as a Cell Target for Genetic Modification and Gene Therapy

Several genetic engineering methods to modify the MSCs gene expression profile have been described, as seen in Table 2. These techniques can be classified as those using viral vectors or non-viral methods. Replication-deficient viruses are the most used gene transfer tools, mainly due to their high efficiency in DNA transfer when compared to non-viral methods. However, the use of viral vectors in the clinical practice has been limited by the high cost of cell line production and the possibility of adverse immunological reactions, or even the occurrence of insertional mutagenesis, which may lead to the activation of oncogenes (Park J.S. et al., 2015).

**TABLE 2:** Genetic engineering methods for MSCs modification.

Genetic engineering methods	Advantages	Limitations
**Retrovirus**	- Easy manipulation and vector design	- Only transduce dividing cells
	- Stable and efficient gene transfer	- Risk of insertional mutagenesis
	- Extensive cell transduction tropism	
**Lentivirus**	- Transduce dividing and non-dividing cells	- Risk of insertional mutagenesis
	- Stable and efficient gene transfer	- Size of the therapeutic gene (<10 kb) insert
	- High levels of transgene expression	
	- High titers	
**Adenovirus**	- Efficient gene transfer	- Transient gene expression
	- Large insert capacity	- High immunogenicity
**Adeno-associated virus**	- Efficient gene transfer	- Small size of the therapeutic gene insert (<5 kb)
	- Extensive cell transduction tropism	- Low yield (hard to produce)
	- Low immunogenicity	- Transient gene expression in dividing cells
**Non**-**viral (Liposomes and plasmids)**	- Low immunogenicity	- Low transfection efficiency
	- Safe manipulation	- Transient gene expression
**CRISPR/Cas9, ZFN and TALEN**	- Precise gene edition (mutation, insertion, replacement, or deletion)	-Off-target effects risk
	- Relative high efficiency	- Target sequencing must precede a protospacer adjacent motif (PAM) (CRISPR/Cas9) -Not easy to design (ZNF and TALEN)

Non-viral methods, on the other hand, can be manufactured on a large scale and have low immunogenicity. Currently, the genetic modification of MSCs using non-viral vectors can be performed by physical or chemical methods. The physical methods used in MSCs include electroporation, nucleofection and sonoporation (Otani et al., 2009; Baraniak and Mcdevitt, 2010; Cantinieaux et al., 2013). Chemical methods use lipidic agents, polymers and inorganic nanoparticles (Uchimura et al., 2007; Park et al., 2010). Although the use of non-viral vectors has some advantages for the process of genetic modification of MSCs compared to viral vectors, the impairment of cell viability, low efficiency and transient expression of transgenes make these methods less used in the clinical practice (Marofi et al., 2017).

Viral vectors are the most used tool in the MSC genetic modification protocols, and it has been demonstrated that the high efficiency of viral transduction in these cells (approximately 90%) does not affect their immunophenotypic characteristics, as well as their potential for cell differentiation and secretion of bioactive molecules, which are preserved after genetic modification (Delcayre et al., 2005; Biancone et al., 2012). In addition, viral transduction ensures stable and long-term transcription of the gene of interest and, consequently, a greater efficiency compared to other methods that do not use viral vectors for genetic modification of MSCs (Sage et al., 2016). Currently, there is extensive clinical experience with several types of vectors that include mainly vaccinia, measles, vesicular stomatitis virus (VSV), polio, reovirus, adenovirus, lentivirus, retrovirus, adeno-associated virus (AAV), and herpes virus simplex (HSV). Among those, the most predominant vectors used for cell transduction and transplantation are the lenti- and retroviral vectors. Beyond those applications, AAV vectors have been used as favored vehicle for direct gene delivery to specific tissues (Finer and Glorioso, 2017).

Adenoviral vectors do not integrate into the host genome and can transduce both dividing and quiescent cells with high efficiency. Obtaining high titers of the recombinant vectors is relatively easy with little cytotoxic effect on the packaging cells (Vemula and Mittal, 2010). However, the high immunogenicity and transient expression of the transgene limits its application in clinical practice (Somia and Verma, 2000). Adeno-associated viruses, on the other hand, have no viral gene in its recombinant form used for gene therapy, a characteristic that contributes to a low immunogenicity and pathogenicity. Also, they are dependent on co-infection with other viruses, mainly adenoviruses, in order to replicate. Recombinant adeno-associated viruses episomal DNA is unable to integrate in the host genome, therefore reducing the consistency of transgene expression along the time in proliferating cells (Sage et al., 2016; Naso et al., 2017). However, a limiting factor for the use of AAVs is the action of neutralizing antibodies present in a large part of the population, which drastically reduces their effectiveness *in vivo* (Nayak and Herzog, 2010).

Retroviruses are RNA viruses made up of three essential *genes*: gag, pol and env, which encode the structural protein, reverse transcriptase / integrase and glycoprotein of the viral envelope, respectively. These genes are arranged in separate plasmids and separately for the packaging cells in order to avoid recombination or generation of retroviruses competent for viral replication. Obtaining high viral titers is relatively easy using these vectors; however, the biggest limitation of retroviral vectors is their inability to transduce quiescent cells. Lentiviruses, however, have the property of transducing dividing and quiescent cells (Lewis et al., 1992; Vargas et al., 2016). Currently, lentiviral vectors are the most used in gene therapy, whether in preclinical or clinical studies, as they guarantee high process efficiency, as shown by the increased number of phase I clinical studies evaluating the safety of gene therapies using lentiviruses conducted in recent years (Milone and O’Doherty, 2018).

Recently, new genetic modification tools arose in order to promote insertion, deletion or correction of genes at specific sites in the genome, and MSCs have been used as a target for these new modifying tools for applications in different diseases (Torres et al., 2014; van den Akker et al., 2016; Gerace et al., 2017; Li et al., 2018; Meng et al., 2019). The site-specific integration of genes can be achieved using tools such as Zinc Finger Nuclease (ZFN), Transcription Activator-Like Effector Nuclease (TALENS) or Clustered Regularly Interspaced Short Palindromic Repeats (CRISPR/Cas9), which are nucleases capable of recognizing and direct the integration of genes in a site-specific manner. These molecular-editing tools can be further explored to improve safety and efficiency of gene insertion/expression (Park J.S. et al., 2015).

## Applications in Cancer Treatment

Cancer is the second leading cause of global death (WHO, 2020), and conventional chemotherapies have shown poor efficacy for the treatment of cancer in advanced stages. Cell therapy emerged in the last years as a promising tool for cancer treatment. Furthermore, a growing number of cell therapies are being tested in combination with other therapeutic agents, such as checkpoint inhibitors (Li et al., 2017; Hu et al., 2018; Cao et al., 2019).

Along the past years, many studies have been focused on MSCs potential to act as “Trojan horses,” promoting the delivery of anticancer immunostimulatory agents, such as chemokines and cytokines, to cancer site due to their tumoral tropism (Hmadcha et al., 2020). The chemokine receptor 4 (CXCR4) plays a critical role in MSCs homing and survival to tumor sites. Overexpression of CXCR4 in adipose tissue (Ad-MSCs) and bone marrow (BMSCs) MSCs promoted anti-tumor activity in different studies (Bobis-Wozowicz et al., 2011). In a mouse model of colitis-associated tumorigenesis, BMSCs transduced with lentiviral vector carrying either CXCR4 had an enhanced homing (2-fold) to inflamed intestinal tissues when compared to control MSCs (Zheng et al., 2019). This higher migratory and engraftment capacity was also observed in an *in vivo* breast cancer mouse model, in which MSCs administered systemically via the tail vein reached the tumors, even though part of the cells remained in the lungs. Moreover, the overexpression of CXCR4 in MSCs increased the accumulation in the tumor compared to non-CXCR4-overexpressing MSCs (Kalimuthu et al., 2017). A study using BMSCs genetically modified to express the CXC receptor 7 (CXCR7), a newly discovered chemokine ligand 12 (CXCL12) receptor that promotes migration to tumor, showed an increase in cell migration and proliferation, which may be attributed to the CXCL12 secreted by MSCs, thus promoting a positive feedback for CXCL12/CXCR7 axis (Liu et al., 2018).

Another explored cancer therapy strategy was the genetic modification of Ad-MSCs to overexpress the cytokine interferon-beta (IFN-β) in an *in vitro* model of canine melanoma. The cytokine family in which IFN-β belongs is known for their antiviral, immunomodulatory and antiproliferative effects. This approach was tested as an adjunctive therapy in order to begin and maintain skin lesion remission caused by pemphigus foliaceus. Ad-MSCs overexpressing IFN-β was associated with pro-apoptotic and growth-inhibitory effects on canine melanoma cells when compared with non-modified MSCs (Han et al., 2015).

The tumor homing of MSCs has been used to deliver TNF-related apoptosis-inducing ligand (TRAIL), known by its anticancer properties. MSCs overexpressing TRAIL (MSC-TRAIL), when co-cultured with tumor cells, induced a 6-fold increase in apoptotic cell death when compared to non-modified MSCs. In addition, these cells were tested in a metastatic lung mouse model, promoting a tumor free rate of 37.5%, whereas no animals were tumor-free in the control MSCs group (Loebinger et al., 2009). Confirmation of the efficacy of MSC-TRAIL *in vivo* were shown by Spano et al. (2019), which tested Ad-MSC-TRAIL in pancreatic cancer, finding a 37% reduction in tumor size in Ad-MSC-TRAIL group compared to non-modified Ad-MSCs, a result which was similar to the that found in the group treated with recombinant TRAIL. Moreover, an increased cytotoxicity of TRAIL-expressing MSCs compared to control MSCs was also seen in a lung cancer *in vivo* model, possibly by enhancing the apoptosis of CLDN7-negative non-small cell lung cancer cells (Xia et al., 2013).

The effects of MSCs-derived exosomes, which are secreted vesicles with an average size of 100 nm originated from the endosomal compartment (Kalluri and LeBleu, 2020), have also been explored in the context of cancer therapy (Gao and Jiang, 2018). Exosomes isolated from MSCs overexpressing microRNAs (MIRs) were also evaluated in different cancer models (Wang B. et al., 2019; Xu et al., 2019; Chen et al., 2020). Treatment with BMSCs-derived exosomes overexpressing miR-16-5p in a mouse model of colorectal cancer (CRC) inhibited invasion, migration and proliferation of malignant cells, and induced a 4-fold higher apoptosis rate *in vitro* compared to control cells (Xu et al., 2019). Exosomes from BMSCs overexpressing miR-34 were 2-fold more potent than control MSCs in inducing the inhibition of invasion, migration, proliferation and tumorigenesis of glioblastoma cells, *in vitro* and *in vivo* (Wang B. et al., 2019). Exosomes from miR-101-3p and miR-199a-overexpressing BMSCs also inhibited invasion, migration and proliferation of oral cancer cells (Xie et al., 2019) and glioma cells (Yu et al., 2019). Furthermore, inhibition of the tumor progression was also observed *in vivo* in a glioma model (Yu et al., 2019).

In a prostate cancer model, administration of BMSCs overexpressing sirtuin 1 led to a decrease in tumor growth and an increase in immune inflammatory response associated with the IFN-γ-induced recruitment and activation of tumoricidal macrophages when compared to non-modified MSCs (Yu Y. et al., 2016). The effects of conditioned medium of umbilical cord-derived MSCs (UC-MSCs) overexpressing hepatocyte nuclear factor 4α (HNF4α), a transcription factor which acts as a master regulator of hepatic differentiation and liver metabolism, has also been evaluated in a mouse model of hepatocellular carcinogenesis (HCC). UC-MSC-HNF4α suppressed metastasis and proliferation of cancer cells when compared to untreated and control MSCs-treated groups. Furthermore, in an *in vitro* assay, MSC-HNF4α reduced approximately 5-fold the migration and invasion potential of cancer cells compared to controls (Wu et al., 2016).

## Applications in Cardiovascular Diseases

Novel treatments for improving the heart function are of immense clinical importance, and cell-based therapies show a great promise (Jadczyk et al., 2017). A variety of cell types, including MSCs, have been used in strategies for inducing cardiac regeneration (Madonna et al., 2010). Initially, it was proposed the differentiation of transplanted MSC into cardiomyocytes and vessels as the main mechanism underlying their therapeutic action in cardiovascular diseases (Tomita et al., 1999; Pittenger and Martin, 2004). However, the number of MSCs-derived cells has been shown to be too low to contribute to the functional improvements, and evidence supports the hypothesis that MSC-mediated paracrine mechanisms play an essential role in tissue repair (Luo et al., 2019). Among the mechanisms promoted by MSCs are neovascularization, cytoprotection, endogenous cardiac regeneration, modulation of inflammatory and fibrogenic processes, cardiac contractility and cardiac metabolism (Gnecchi and Cervio, 2013).

Mesenchymal stem/stromal cells have been genetically modified to express factors that resulted in significant improvements in cardiac recovery. Several authors selected factors to be introduced in a gene therapy approach, based on the putative beneficial effects of MSCs described in infarct myocardial models, such as growth factors. MSCs overexpressing vascular endothelial growth factor (VEGF), when delivered by intramyocardial injection, reduced the infarcted area in 31% and improved left ventricular function to almost its baseline when compared with non-transfected MSCs (Locatelli et al., 2015). In a mouse model of myocardial infarction, transplantation of umbilical cord-MSCs overexpressing hepatocyte growth factor (HGF), a pro-regenerative factor, reduced by approximately half the infarcted area when compared with non-transduced cells, in addition to significantly increase the survival rate post-transplant (Zhao et al., 2016). In addition, the pro-survival cytokine insulin-like growth factor (IGF)-1, when overexpressed by BMSCs, reduced by approximately 50% the number of apoptotic myocardial cells post-transplant compared to animals treated with control MSCs, and increased MSC retention in the tissue in an experimental model of myocardial infarction (Haider et al., 2008). Similar results were observed after C1q/tumor necrosis factor-related protein-3 (CTRP3) and CCR1-modified MSCs transplant. MSCs overexpressing CTRP3, which is associated with protective effects after myocardial infarct, had an enhanced survival and retention 7 days post-transplant, approximately three times higher when compared to unmodified control cells (Zhang Z. et al., 2019). Additionally, overexpression of CCR1 increased by ∼90% the chemokinesis of MSC *in vitro* and *in vivo*, also increasing by more than 50% the amount of viable MSCs in the infarcted myocardium area (Huang et al., 2010).

The therapeutic effects of MSCs overexpression growth factors were also studied in a mouse model of cardiomyopathy induced by chronic infection with *Trypanosoma cruzi*, the causative agent of Chagas disease. Administration of genetically modified mesenchymal cells overexpressing granulocyte colony-stimulating factor (G-CSF) were about 2-fold more potent in reducing the number of inflammatory cells and the percentage of fibrosis than control MSCs, acting by increasing the number of myeloid-derived suppressor cells and T regulatory cells (Silva et al., 2018b). In contrast, no increase in anti-inflammatory or antifibrotic activity was seen after transplantation of IGF-overexpressing MSCs in *T. cruzi*-infected animals. However, an increased pro-regenerative activity in skeletal muscle was observed after MSCs-IGF-1 transplantation, compared to control MSCs, increasing the number of myofibers similar to that of uninfected mice (Silva et al., 2018a).

The overexpression of the regulators of cardiogenesis Csx/Nkx 2.5 and GATA-4 was also investigated in a myocardial infarct model. Genetically modified MSCs overexpressing these factors increased the cardiac function by inducing pro-angiogenic effects, significantly increasing microvessel density in the peri-infarct regions and reducing cell loss by apoptosis by approximately 10% compared to animals treated with control MSCs (Gao et al., 2011; He et al., 2019).

In addition to their pro-angiogenic effects, the cellular benefits of MSCs may also be mediated by activating the survival kinase pathways, including Akt activation, in cardiomyocytes in response to MSC-secreted cytokines, promoting a reduction programmed cell death (Jiang et al., 2013). Interestingly, MSCs overexpressing Akt promoted the maintenance of metabolism, glucose uptake and cytosolic pH and prevention of cardiac metabolism remodeling in a myocardial infarct model. Remarkably, for Akt-MSC hearts, systolic performance was only 12% (72 h) to 17% (2 week) lower compared with sham-operated hearts while diastolic performance remained normal. In contrast, for unmodified MSC-treated and untreated infarcted hearts, markers of both systolic and diastolic performance were significantly lower than for sham-operated hearts (Gnecchi et al., 2009). Akt-modified amniotic fluid derived MSCs were also capable of alleviating myocardial injury and contribute to cardiac regeneration in an ischemia-reperfusion injury in a rabbit model, promoting angiogenesis by capillary density enhancement and VEGF expression 2-fold more than control MSCs. Moreover, Akt-expressing MSCs promoted a significant increase in cTNT, GATA-4 and connexin 43 compared to controls (Wang et al., 2016). In addition, purified exosomes from TIMP2- and Akt-modified umbilical cord MSCs promoted improvements in cardiac function, by activating TIMP/Akt pathway (Ni et al., 2019) and increasing platelet-derived growth factor D expression (Ma et al., 2017), in rats submitted to myocardial infarction.

The expression of CXCR4, the stromal cell-derived factor (SDF)-1 receptor, which is largely involved in progenitor homing and survival, has been tested to improve the insufficient cell homing and tissue persistence observed in several preclinical studies. CXCR4-MSCs had a 3-fold increase in cell migration capacity *in vitro* and *in vivo* in a myocardial infarct model when compared to control MSCs, correlating with an increased expression of metalloproteinases. Additionally, a 4-fold increase in capillary density was found in the hearts of animals treated with CXCR4-MSCs when compared to control MSCs groups (Huang Z. Y. et al., 2012). A significant improvement in cardiac function in CXCR4-MSCs treated animals compared to those treated with control MSCs, which may be explained by the reduction of heart fibrosis and increased angiogenesis post-injury (Huang W. et al., 2012; Mayorga et al., 2017; Wu S. Z. et al., 2017).

Furthermore, anti-inflammatory interleukins and regulators of oxidative factors were explored in the production of genetically engineered MSCs. IL-10 overexpression improved the therapeutic effects of MSCs by reducing by 2.5-fold the production of proinflammatory cytokines (TNF and IL-1β) and promoting a 3-fold reduction of the heart infarct size when compared to control MSCs (Meng et al., 2017, 2019). In addition, injection of MSCs modified to overexpress IL-33, a cytokine known to induce Th2 and Treg responses (Schmitz et al., 2005) resulted in a significant improvement of heart function and reduced inflammation compared to vector control MSCs in rats with myocardial infarction (Chen et al., 2019). MSCs modified to overexpress endothelial nitric oxide synthase (eNOS) were more potent than control MSCs and eNOS adenoviral vector in reducing the myocardial infarct size (4 and 2-fold increase, respectively), corrected hemodynamic parameters and increased the capillary density by increasing nitric oxide production (Chen L. et al., 2017). Moreover, heme oxygenase-1 (HO-1), an enzyme acting on heme degradation, as well as in the generation of cytoprotective agents (Liu and Qian, 2015). Transplantation of HO-1-overexpressing MSCs promoted not only an improvement in angiogenesis in scar areas, but also an increase in connexin 43-positive gap junctions and a higher tyrosine hydroxylase-positive cardiac sympathetic nerves sprouting in a myocardial infarction model (Zeng et al., 2008, 2010; Yang et al., 2012).

Other molecules with diverse mechanisms of action were also explored as modification targets were Follistatin-like 1 and Islet-1. MSCs overexpressing Follistatin-like 1, a pro-survival cardiokine for cardiomyocytes, had increased survival, proliferation and engraftment, thereby improving in about 2-fold their therapeutic efficacy when compared to mcherry transgenic control MSCs in a myocardial infarction model (Shen et al., 2019). Similarly, overexpression of Islet-1, a transcription factor involved in cardiogenesis regulation and a marker of cardiovascular progenitor cells, promoted MSCs survival post-transplant and enhanced their paracrine function (Xiang et al., 2018).

Another strategy studied to improve cell therapies for cardiac diseases is the overexpression of microRNAs in MSCs. Overexpression of mIR-126 in MSCs induced the production of Notch ligand Delta-like-4, which activates the Notch pathway and promotes the production of pro-angiogenic factors, ameliorating the cell therapy efficacy in infarcted hearts by 2-fold compared to control MSCs (Huang et al., 2013b). In a similar way, intramyocardial transplantation of microRNA-1-transfected MSCs was more effective to promote repair of the infarct injury and improvement in heart function by enhancing transplanted cells survival and cardiomyogenic differentiation when compared to mock-transfected MSCs (Huang et al., 2013a). Furthermore, in a model of myocardial ischemic-reperfusion injury, mIR-181a overexpression enhanced the immunosuppressive capacity of MSCs-derived exosomes, improving their therapeutic effect by 2 to 3-fold in reducing the infarct area and increasing the ejection fraction when compared to exosomes produced by control MSCs (Wei et al., 2019). Genetic modification of MSCs to overexpress miR-21 not only promoted an enhanced migration and proliferation rates, but also in angiogenesis and cardiac function. This may be explained by an increase in Bcl-2, Cx43 and VEGF levels and a decrease in Bax, BNP and troponin T, seen in a model of adriamycin-induced myocardial damage (Zeng et al., 2017). Transplantation of MSCs overexpressing miR-133 in a rat model of myocardial infarction reduced fibrosis and promoted an improvement in cardiac function more potently than control vector-MSCs, possibly by reducing *Snail 1* expression (Chen Y. et al., 2017). Finally, in a model of myocardial infarction, intravenous injection of MSCs genetically engineered to overexpress miR-211, known to influence cell migration, promoted a significant increase in migration of MSCs to the injured area and reduction of the infarct size, while PBS or control MSCs did not reduce the infarct size nor promoted functional recovery (Hu et al., 2016).

## Applications in Lung Diseases

Pulmonary tract pathologies are commonly due to the frequent exposure of the respiratory system to different factors, such as tobacco, toxic smoke or polluted air, which may contribute to the development of acute and chronic disorders, such as infections or autoimmune and inflammatory diseases. Thus, MSCs have been used and genetically modified to express different factors aiming to promote the patients’ recovery, since many diseases still do not have an efficient treatment.

Pulmonary arterial hypertension (PAH) has been a target of therapies with modified mesenchymal cells. Guo and collaborators generated rat bone marrow derived MSCs expressing human HGF (MSC-HGF) by transduction with recombinant adenoviral vector (ad-HGF). The treatment with MSC-HGF or MSC-HGF+ recombinant G-CSF promoted a significant reduction in mean pulmonary arterial pressure and hypertrophy in the right ventricle when compared with the control MSCs and untreated groups. In addition, pulmonary perfusion in the MSC-HGF+G-CSF group was improved by increasing the number of blood vessels (∼2-fold more vessels compared with the MSC and untreated group) (Guo et al., 2013). Treatment with MSCs overexpressing the secreted Klotho protein (SKL), a β-glucuronidase that regulates oxidative stress and inflammation, restored pulmonary endothelial dysfunction in a model of monocrotaline-induced PAH, acting by reducing 25% blood vessel thickness and doubling the lumen when compared to the MSCs, MSCs-GFP and untreated groups. MSCs-SKL also slightly attenuated systolic pressure and right ventricular hypertrophy and had anti-inflammatory effect, reducing by 50% the macrophage infiltration in the lung tissue (Varshney et al., 2016).

In a severe acute lung injury (ALI) mouse model, treatment with bone marrow-derived MSCs modified to express human angiopoietin 1 (Ang1) promoted a significant reduction in airspace inflammation, with 96 % less neutrophils than groups treated with control MSCs or saline. Moreover, Ang 1-MSCs reduced the production of pro-inflammatory cytokines IFN-γ, TNF-α, IL-6 and IL-1β, equal to baseline values observed in naive mice (Mei et al., 2007). In a similar study, MSCs transduced by lentiviral vector to overexpress angiopoietin 1 (MSC-Ang1) also promoted benefits for ALI, reducing the levels of the pro-inflammatory cytokines TGF-β1 and IL-1β and increasing the expression of the anti-inflammatory cytokine IL-10 in the serum and bronchoalveolar lavage fluid, in addition to improving the lung function by increasing the expression of surfactant protein C (Shao et al., 2018).

*In vitro* studies with alveolar epithelial cells subjected to cigarette smoke extract (CSE) revealed that BMSCs overexpressing Notch1 receptor (MSC-N1ICD) maintained alveolar cells proliferation levels close to those observed in the control not exposed to CSE by activation of PI3K/Akt pathway. Additionally, MSC-N1ICD doubled the expression of CXCR4, promoted a 2-fold greater cell migration when compared to the control MSC groups and prevented apoptosis, as shown by the 50% reduction in caspase-3 expression in alveolar cells when compared to MSC control cells (Cheng et al., 2017). In another study, Cai et al. (2015) reported that MSCs genetically modified to express Wnt/β-catenin contributed *in vivo* with the alveolar epithelium protection, promoting the retention of a greater number of MSCs in the lungs for up to 14 days when compared to the control cell line, as well as an increase (approximately 3-fold) of differentiation of MSCs in type II alveolar epithelial cells and improvements in alveolar epithelial permeability, resulting in a reduction in lung damage.

Mesenchymal stem/stromal cells from different sources have been genetically modified to express different cytokines and chemokines in order to explore the immunomodulatory potential of these cells in lung injury models. The studies demonstrated that MSCs overexpressing IL-10, CXCR4 or CXCR7 caused a decrease in the number of alveolar neutrophils and levels pro-inflammatory cytokines, such as tumor necrosis factor α (TNFα), transforming growth factor (TGF-β1) and IL-6. Moreover, an increase in the survival rate of the animals treated with these genetically modified cells was observed, likely due to the reduction of inflammatory severity and damage to the lungs (Wang et al., 2018; Jerkic et al., 2019; Shao et al., 2019; Zhang C. X. et al., 2019). Differentiation of MSCs into type II alveolar epithelial cells and an increase of alveolar macrophages were also observed (Jerkic et al., 2019; Shao et al., 2019).

Overexpression of enzymes and receptors by MSCs was another approach used in the management of ALI. In a model of LPS-induced lung injury, mouse BMSCs modified by a lentiviral vector to express the angiotensin-converting enzyme 2 (ACE2) induced an improvement in the lung tissue, with decreased inflammatory infiltrate, edema and interstitial hemorrhage and reduction of lung injury score from 13 to 5.7 in WT mice, and to 8.6 in ACE-knockout mice when compared to the MSC and untreated groups. In addition, a decrease in IL-6 and IL-1β levels, as well as an increase in IL-10 levels, was observed in the MSC-ACE2 group compared to the control MSCs (He et al., 2015). Similarly, Martínez-González et al. (2013, 2014) observed that human MSCs derived from adipose tissue overexpressing the soluble IL-1 receptor type 1 (sST2) caused a decrease in histopathological changes associated with lower levels of pro-inflammatory cytokines TNF-α, IL-6, and MIP-2 (1.5 to 3-fold reduction), and 6-fold higher levels of IL-10 in models of ALI and asthma. In a model of ALI in rats, BMSCs overexpressing the enzyme heme-oxygenase-1 (HO-1) promoted the survival and anti-apoptotic activity greater than wild-type BMSCs (Chen X. et al., 2018). The transplantation of MSCs overexpressing the type 2 angiotensin II receptor (AT2R) increased cell migration *in vitro* by 3-fold, inhibited inflammation by decreasing pro-inflammatory cytokines (IL-1β and IL-6) and recovered the injured lung in a model of ALI in mice (Xu et al., 2018), while overexpression of the orphan receptor tyrosine kinase 2 (ROR2) by MSCs not only improved their ability to relieve inflammation and histopathological changes, but also increased their retention in lungs (Cai et al., 2016).

Exosomes derived from MSCs are important sources of microRNAs that provide protection against various diseases. Therefore, increasing the expression of microRNAs in MSC is an interesting alternative for some pulmonary pathologies. In a study that used exosomes derived from MSCs with overexpression of miR-30b-3p in co-culture with alveolar epithelial cells (AECs) challenged with type II LPS, high endocytosis of exosomes rich in miR-30b-3p was found, and this phenomenon was accompanied by reduced expression of SAA3 (protein highly expressed in ALI), increased cell proliferation and inhibition of apoptosis (Yi et al., 2019). BMSCs overexpressing let-7d, an antifibrotic microRNA, were tested in an ALI model in mice, promoting an increase in the production of the anti-inflammatory cytokine IL-1RN (when compared to wild type MSC), a reduction in the number of CD45^+^ cells (when compared to the untreated group) and decreased collagen transcription levels, even though no significant differences were observed in the Ashcroft score and OH-proline (Huleihel et al., 2017). UC-MSCs overexpressing p130 or E2F4 also proved to be more potent in decreasing tissue damage in the lungs, promoting retention of MSC-p130 and MSC-E2F4 7 days after intratracheal transplantation (50% more retained cells than control groups) and inhibiting fibrosis in a model of severe acute respiratory distress (Zhang X. et al., 2019).

Transcription factors are important regulators of gene expression. The effects of human amniotic MSCs (hAMSCs) modified to express the nuclear factor erythroid-derived 2-like 2 (Nrf2), a transcription factor that regulates the expression of genes related to antioxidant activity, was evaluated in a mouse model of lung injury induced by intratracheal LPS instillation. Nrf2-overexpressing cells were about 2-fold more potent than control MSCs in reducing apoptosis, fibrosis, edema and pro-inflammatory cytokine levels when injected intravenously (Zhang et al., 2017).

## Applications in Gastrointestinal and Liver Diseases

Genetic modification of MSCs is a promising approach widely explored in diverse models of gastrointestinal and liver diseases. The improvement in cell homing and target ability to inflammatory sites, together with the increase in immunosuppression and tissue repair capacity after transplant, are some of the improvements seen in gastrointestinal experimental models of oral mucositis and inflammatory intestinal diseases.

Growth factors have been used as targets for gene therapy in the field of liver disease, as shown in a study in which HGF-overexpressing BMSCs (BMSC-HGF) promoted an improved recovery from liver damage in a model of CCl_4_-induced cirrhosis in rats, increasing the expression of hepatic proteins HNF-4α, CK18 and ALB (2 to 3.5-fold greater than untreated control) and reducing the presence of liver injury markers, aspartate aminotransferase (AST), alanine aminotransferase (ALT), and total bilirubin, compared to the other groups tested (Zhang Y. et al., 2018). The therapeutic effects of HGF overexpressing MSCs were also studied in an acute model of radiation-induced liver injury. MSC-HGF showed greater migration and permanence in the injured tissue, increased the proliferation of hepatocytes (about 4-fold greater than untreated control), completely blocked the increase in ALT and AST and prevented apoptosis and liver fibrosis when compared with the other treatments (Zhang J. et al., 2014). In a similar model of chronic injury, MSCs overexpressing fibroblast growth factor 4 (FGF4) also contributed with liver regeneration, promoting greater migration of MSC-FGF4 to cirrhotic livers and increasing the expression of the liver progenitor marker EpCAM by about 4-fold when compared to control groups (Wang et al., 2015).

Hepatic macrophages (Kupffer cells) play a central role in liver fibrosis, being critical in both its promotion and resolution. In a model of chronic thioacetamide poisoning (TAA), MSCs transduced with a lentiviral vector for overexpression of IGF-1 (Ad-MSC-IGF-I) were able to reverse a pro-fibrotic phenotype in Kupffer cells, reducing by half the levels of collagen deposition in the area of the lesion when compared to the vehicle-treated group and downregulating the expression of the pro-fibrogenic genes TGF-β1, α-SMA and collagen 1A2 (COL1A2) (Fiore et al., 2015). Neuregulin 4 (Nrg4) acts in the liver, where it modulates the lipogenesis in hepatocytes by activating the ErbB3/ErbB4 signaling. Thus, the therapeutic potential of Nrg4-overexpressing Ad-MSCs was tested in a model of hepatic steatosis induced by a high-fat diet. Transplantation of MSC-Nrg4 reduced weight gain, decreased serum glucose and insulin levels, in addition to improving glucose intolerance more significantly than treatment with control MSC (Wang W. et al., 2019).

Cytokines and chemokines have also been evaluated in liver and gastrointestinal diseases. Uncontrolled hepatic immune activation is the primary pathological mechanism of fulminant hepatic failure (FHF). The interleukin-1 receptor antagonist (IL-1Ra) plays an anti-inflammatory and anti-apoptotic role in acute and chronic inflammation. The transplantation of MSCs that overexpress this molecule increased survival (63.6% survival in the MSC-IL-1Ra group versus 30.0% and 22.2% in the MSC and vehicle groups). MSC-IL-1Ra increased the fraction of proliferating hepatocytes (23.5%) compared to MSC and control (19.9% and 18.32%, respectively), and improved liver function by negatively regulating inflammatory responses activated by IL-1 *in vivo* (Zheng et al., 2012). In a TAA-induced model of chronic liver fibrosis in mice, engineered MSCs that secrete high levels of IL-10 had a more potent antifibrotic activity and caused the improvement in liver function than control MSCs, which were also able to significantly promote histopathological and functional improvements (Choi et al., 2019).

The role of CXCL2 in oral mucositis was investigated from transplantation of human BMSCs with increased expression of CXCR2 by lentiviral transduction. MSC-CXCR2 exhibited improved ability to migrate in response to CXCL2 *in vitro* (almost 2 times greater than MSC control), indicating greater targeting of these cells to the inflamed mucosa in animal models. MSC-CXCR2 also had a longer residence time in the oral cavity than control MSC and promoted accelerated ulcer healing by reducing pro-inflammatory cytokines, such as TNF-α, IL-1β, and IL-6 and levels of reactive oxygen species (ROS) in epithelial cells were 2-fold lower, while ROS levels were 4-fold lower in tongue fibroblasts (Shen et al., 2018). In a mouse model of inflammatory bowel disease (IBD) induced by sodium dextran sulfate, increased expression of the Intercellular Adhesion Molecule (ICAM)-1 by MSCs have been shown to alleviate inflammatory damage in IBD mice, reducing colon shortening to levels closer to the control without IBD, in addition to halving the number of inflammatory cells when compared to the untreated IBD group. Moreover, ICAM-1 also stimulated the migration of modified MSC to the affected colon (Li et al., 2019).

In addition, the transfer of genes coding for important transcription factors regulating hepatogenesis, such as HNF4α and forkhead box protein (Foxa2), was investigated in the context of liver diseases in association with stem cell therapy. MSCs overexpressing Foxa2, cultured in a 3D system of poly-lactic-co-glycolic acid (PLGA) scaffold, promoted hepatic differentiation, *in vitro* and *in vivo* when implanted into nude mice. Importantly, when implanted into dorsal subcutaneous tissues, Foxa2-overexpressing Ad-MSCs/scaffolds reduced the alterations in liver function markers in the blood and in liver tissue, in an acute liver injury model induced by TAA (Chae et al., 2019). Moreover, Foxa2-MSCs promoted the recovery expressions of liver enzymes close to normal levels, and reduced the fibrotic areas more potently when compared to MSC alone or MSC/vector groups (Cho J.W. et al., 2012). Similarly, HNF-4α-modified MSCs showed increased therapeutic effects in a liver cirrhosis mouse model, where MSC-HNF-4α positively regulated iNOS expression by activating the NF-κB signaling pathway (Ye et al., 2019).

## Applications in Kidney Diseases

Nephrological disorders can lead to an increase in mortality and morbidity, especially in cases of acute kidney injury, and may affect the functionality of the organs. The use of genetically modified MSCs has also been investigated in the context of kidney diseases.

Ad-MSCs and BMSCs genetically engineered to overexpress glial cell line-derived neurotrophic factor (GDNF) promoted the switching of macrophages to a reparative phenotype, reduced renal fibrosis and contributed to a recovery in renal function in models of nephrotoxic serum nephritis and unilateral ureteral obstruction (Huang Z. Y. et al., 2012; Wang Z. et al., 2019). In another study, injection of MSC-HGF, in a model of bladder outlet obstruction, not only prevented collagen deposition, with a reduction in collagen area of almost half when compared to the untreated animals, as well as increased cystometric parameters in transplanted animals (Song et al., 2012). In a model of renal failure-induced anemia, MSC-IGF-1 co-implanted with MSCs genetically engineered to secrete erythropoietin (MSC-EPO) promoted a significant hematocrit elevation when compared to the group which received the MSC-EPO + MSC-null, with a difference of approximately 20% after 98 days. Additionally, an improvement in heart function was observed in the animals treated with MSC-EPO + MSC-IGF-1 (Kucic et al., 2008).

Overexpression of cytokines and chemokines in MSCs have also been evaluated in models of kidney diseases. In a model of renal ischemia/reperfusion injury, MSCs genetically modified to overexpress TGF-β1 increased renal function and reduced inflammation by a significant decrease in the pro-inflammatory cytokine expression, such as IL-2, IL-6, and TNF-α, in comparison to the PBS and MSC groups, and an enhancement in IL-10 expression of about 2.5-fold when compared to the sham group (Cai et al., 2019). Lipocalin 2 (Len2), secreted in high levels into the blood and urine after kidney injury may be an important cytoprotective agent against injuries caused by oxidative stress (Roudkenar et al., 2008, 2011). BMSCs overexpressing Len2 prevented cytotoxicity and apoptosis when co-cultured with HK-2 and HEK293 cells in a model of cisplatin-induced kidney injury *in vitro*, with a cell viability of 60% after 70 days when co-cultured with HK-2 cells and 40% with HEK293 cells, while the control groups with only kidney cells co-cultured with MSCs or Mock-MSCs had 20% and less than 10% viability for kidney cells, respectively. Additionally, the percentage of apoptosis in the groups treated with MSC-Len2 was significantly decreased, while HGF, IGF-1 and FGF-2 concentrations were higher in this group when compared to the other treatments (Halabian et al., 2014). BMSCs-CXCR4, were also able to stimulate HGF production when compared to the MSCs alone, although not in the same intensity as the MSC-Len2. Furthermore, an increase in IL-10 and bone morphogenetic protein 7 (BMP-7) was also observed. Increasing in proliferating cells and a decrease in apoptotic cell markers when co-cultured with hypoxia/reoxygenation-pretreated renal tubular epithelial cells (HR-RTECs), as well as renal functional improvement and a decrease in tubular cell death was seen in animals treated with BMSCs-CXCR4 in a model of acute kidney injury (Liu et al., 2013).

Transplantation of BMSCs modified to express the Klotho gene, which is known to be related with the aging process (Kuro-o et al., 1997), promoted antifibrotic effects and led to an increase in proliferation and immuno-regulatory ability of MSCs in a model of acute kidney injury (Zhang F. et al., 2018). Moreover, in a model of ischemia/reperfusion injury, administration of exosomes derived from BMSCs expressing miR-199a-3p had protective effects and decreased inflammatory mediators, as well as the number of apoptotic cells, although this reduction was similar to the group treated with the MSCs (Zhu et al., 2019).

## Applications in Neurological Disorders

Due to the complexity of the nervous system, many neurological disorders do not have an effective treatment in order to reduce or prevent the damages caused in the nervous tissue. However, MSC cell therapy has been investigated as an option for the treatment of several neurological disorders, and many articles have studied their effects on the nervous system and possible strategies to enhance their therapeutic actions, including genetic engineering.

Mesenchymal stem/stromal cells secrete diverse growth factors related to neurogenesis and neuroprotection, such as brain derived neurotrophic factor (BDNF), Glial cell-derived neurotrophic factor (GDNF), fibroblast growth factor-2 (FGF-2), HGF, IGF-1, nerve growth factor (NGF) and platelet-derived growth factor (PDGF) (Joyce et al., 2010) and, therefore, many studies have been evaluating the effects of MSCs overexpressing these factors for the treatment of neurological disorders in different *in vitro* and *in vivo* models. In a model of diabetic cistopathy, rats that received intrathecal injections of human MSCs derived from umbilical cord modified by lentivirus to overexpress NGF (MSCs-NGF), had a significant improvement in voiding dysfunction, with a 12% and 45% voiding interval higher when compared to the MSC control and untreated diabetic groups, respectively. This effect was related to the differentiation of MSCs-NGF in neurons and glial cells, increasing the control of neuronal functions along the urinary pathway (WenBo et al., 2017). In the *in vitro* model of Parkinson’s disease, the conditioned medium MSCs overexpressing HGF showed an increase in the viability of neuron culture 48 h after treatment (about 14% greater than control MSC and 42% greater than untreated cells). In addition, the presence of intracellular free calcium was 2.4-fold lower in the MSC-HGF group and about 2-fold lower in the MSC group when compared to the untreated control, indicating a greater ability of MSC-HGF to maintain the cellular homeostasis (Liu et al., 2014). Supernatant of MSCs overexpressing BDNF from three different donors showed a higher neuroprotective effect *in vitro*, with a neuronal survival rate around 40% when compared to the control-vector infection group (Scheper et al., 2019), while in another study, MSCs also overexpressing BDNF started to express neuronal phenotype markers 1 day after transfection and in the following days, presented a neuron-like morphology when compared to the MSC control, which maintained their fibroblast-like morphology (Lim et al., 2011). Interestingly, these findings demonstrate that the overexpression of BDNF by MSCs seems to be able to either increase their neuroprotective effects or induce changes in their phenotype to one closer to a neuronal one, suggesting more than one possible effect in the cells.

In another study, MSCs with GDNF overexpression increased the differentiation of neural stem cells (NSCs) when these cells were co-cultured with a 3D microfluidic system. After 5 days in culture, a significant increase in the expression of neuronal markers, such as Tuj1 and MAP2, and in the formation of neurites in NSCs co-cultured with MSC-GDNF when compared to MSC control group was observed. In addition, the authors performed an animal model of hypoxic-ischemic stroke to assess neurobehavioral function. However, even demonstrating promising effects *in vitro*, transplantation of MSC-GDNF did not demonstrate a significant functional increase when compared to control MSC, being superior only to the untreated group (Yang et al., 2015). On the other hand, transplantation of MSCs with increased expression of FGF21 (an important regulator of metabolic pathways) in the brain of mice after traumatic brain injury (TBI) led to a decrease in memory deficits and increased the level of FGF21 in the ipsilateral hippocampus, naturally decreased after TBI. In addition, the MSC-FGF21 group had enhanced neurogenesis and restored the morphology of immature newborn neurons in the hippocampus when compared to the MSC and vehicle groups, which exhibited impaired maturation and deficiency in the neurogenesis process (Shahror et al., 2019).

In a model of spinal cord injury (SCI), bone marrow-derived MSCs overexpressing or not ciliary neurotrophic factor (CNTF) promoted functional recovery, as shown by an improvement in the Basso, Beattie, and Bresnahan (BBB) motor score during the weeks following transplantation, and the H&E staining of the injury site demonstrated that both groups showed a greater cell density in the cavity when compared to untreated group. Nevertheless, even with both cell groups seeming to be able to preserve the nervous tissue, the BMSC-CNTF group displayed a profile more similar to the sham group in comparison to the others (Abbaszadeh et al., 2015). Another study evaluating the effects of MSCs on SCI showed that transplantation of MSCs overexpressing HGF reduced the activation of astrocytes by inhibiting the expression of TGFβ1 and β2, promoting an almost 3-fold decrease in GFAP+ cells in the lesion area, when compared to the control or wild type MSCs, in addition to inducing the reduction of the glial scar, one of the main factors that hinder the functional recovery after SCI (Jeong et al., 2012). BMSCs genetically modified to overexpress the growth factor IGF-1 showed promising effects on neuroprotective and anti-oxidant activity in mice after SCI. When compared to control groups, IGF-1 secretion promoted a 3-fold increase in graft survival in the lesion area, which facilitated the recruitment of endogenous neural progenitor cells, as well as the positive regulation of antioxidant genes, resulting better preservation of neural tissue (myelinated area about 50% greater than that observed in control groups) and significant motor improvement using Basso Mouse Scale (BMS; 6 at the end of the experiment for IGF-1 group vs 4 in control groups) (Allahdadi et al., 2019). In another SCI model, BMSCs overexpressing sonic hedgehog (Shh), a growth factor with multiple functions in the nervous system, were able to reduce tissue damage, with 2 times greater expression of neurofilament 200 (NF200), in addition to promoting functional recovery significant in animals when compared to BMSCs or vehicle groups (Jia et al., 2014). Finally, mice treated with BMSCs genetically modified with brain dopamine factor (CDNF) showed significant functional gains in the BBB score, from week 3 to week 6, when compared to control groups, which did not show significant differences between themselves. In addition, histopathological analyzes indicated greater tissue preservation, with a 6 to 7-fold reduction in the area of cavitary lesions in the MSC-CDNF group when compared to MSC control and untreated groups. Neural fiber recovery was also observed, with improvements in remyelination levels and reduction of neuroinflammation after SCI (Zhao et al., 2014).

A study evaluating the effects of neurotrophin-3 (NT3) overexpression by BMSCs in a model of Parkinson’s disease revealed that genetically modified cells protected neuronal tissue and induced differentiation of BMSCs into cells similar to dopaminergic neurons (neuron type affected by Parkinson’s disease), as seen by a 5-fold increase in the levels of nurr-1 and wnt-1 in transfected cells, when compared to treatment with wild-type MSCs (Moradian et al., 2017).

VEGF-overexpressing MSCs have been used in different disease models, including neurological diseases. In the treatment of peripheral nerve damage, MSC-VEGF has shown promising results both *in vitro* and *in vivo*, promoting the extension of neurites and maintaining high expression of VEGF in the nerves 2 weeks after grafting (Man et al., 2016). In a model of Alzheimer’s disease, also evaluating the effects of MSC-VEGF, genetically modified cells promoted neovascularization in the hippocampus and reduced the presence of beta-amyloid plaques in the dentate gyrus when compared to the vehicle-treated group, although no significant differences were found compared to the MSC group, which also appeared to induce neovascularization (Garcia et al., 2014).

The immunomodulatory effects of genetically modified MSCs to express interleukins have been studied to treat autoimmune diseases, such as multiple sclerosis, or to reduce the damage caused by trauma to the brain or spinal cord in cases of TBI, SCI or stroke. Genetically modified MSCs to express IL-4 (responsible for modulating the autoimmune inflammatory response), for example, exhibited protective effects in a model of multiple sclerosis when transplanted in the early stage of the disease. MSC-IL-4 reduced the expression of pro-inflammatory cytokines such as IFNγ and IL-6, leading to a reduction in disease severity when compared to control groups (Payne et al., 2012). In an acute ischemic stroke model, BMSCs overexpressing IL-10 ameliorate the motor function, posture score and forelimb grip strength. In addition, 72 h after treatment, a decrease in the number of Iba-1 and TNF-α positive cells, of approximately 2-fold more, was seen in BMSC-IL-10 group when compared to the vehicle, while this reduction was of almost half in comparison to the MSC, which also presented a significant difference in relation to the vehicle group (Nakajima et al., 2017). The effects of BMSC-IL-10 were also evaluated in a TBI model, in which these cells and MSCs alone decreased significantly the expression of cell death markers, such as Bax, cytochrome-C, caspase-3 and p53 when compared to the vehicle group and increase in those related to cell survival (Bcl2) and synaptic transmission (PSD95 and synaptophysin) was seen in BMSC-IL-10, but not in MSCs alone and vehicle groups, which presented differences only in synaptophysin levels. Thus, these results showed the promising effects of BMSC-IL-10 in promoting the protection of neuronal cells following TBI (Maiti et al., 2019). C-C Motif Chemokine Receptor 2 (CCR2) is positively regulated in the first 24 h after ischemic stroke. Therefore, the effects of MSCs genetically modified to express CCR2 were evaluated in a stroke model. In this study, overexpression of CCR2 increased 5-fold approximately the migration of MSCs modified to ischemic region, and it was able to not only decrease ischemic injuries and promote neurological recovery *in vivo*, but demonstrated an important role in protecting the blood-brain barrier and in promoting endothelial regeneration, when compared to the control groups of MSC (Huang Y. et al., 2018).

Overexpression of enzymes and receptors by MSCs, in order to improve the protective effects in tissue regeneration or increase cell survival, has also been studied for the treatment of different neurological disorders. MSCs derived from different sources have been genetically modified to express important enzymes for the CNS. MSCs overexpressing arginine decarboxylase (an enzyme that regulates the synthesis of agmatine, known to confer neuroprotection after brain damage) were tested in an SCI model in rats. Tissue analyzes showed an increase in BDNF expression at the lesion site, and this effect was accompanied by a reduction in the area of fibrotic scar and functional improvement in the group treated with the modified cells. MSCs overexpressing heme oxygenase-1 (an enzyme that modulates the response to oxidative stress after spinal cord trauma) were tested in canine SCI. Overexpression of this molecule led to a functional recovery after transplantation, related to the anti-oxidative effect and to a decrease in the presence of infiltrated cells, fibroblast-like cells and apoptotic cells in the area of the lesion, as well as an increase in the expression of NeuN and β3-tubulin markers when compared with the control groups. In addition, MSCs with increased tropomyosin kinase A (TrkA) receptor, a receptor highly expressed in sensory fibers, were used to repair peripheral nerves in rats. 8 weeks after transplantation, the MSC-TrkA-treated group showed greater axonal growth, with significantly higher expression of the basic myelin protein and superior results of the density of myelinated fibers, in addition to superior functional performance than observed in the control groups (Park Y.M. et al., 2015; Lee et al., 2017; Zheng et al., 2017).

In another study, MSCs derived from Wharton’s jelly genetically modified to express PARKIN (an ubiquitin ligase capable of protecting dopaminergic neurons against stress) were tested in the Parkinson’s disease model produced by 6-hydroxydopamine-induced toxicity. The overexpression of PARKIN in MSCs was able to significantly reduce the expression of markers of cell death and oxidative stress, in addition to significantly reducing the production of reactive oxygen species (∼ 50% reduction) when compared to wild type and control groups (Bonilla-Porras et al., 2018). Studies in stroke models showed that MSCs overexpressing extracellular regulating kinase 1/2 or integrin ɑ4 not only were able to show induction of neuronal differentiation, proliferation of neural stem cells and a significant functional recovery in mice after the treatment (Gao et al., 2019), but also demonstrated a significant decrease in cell aggregation and improvement of cerebral embolism in rats (Cui et al., 2017).

MicroRNAs have been receiving attention during the past years due to their involvement in the regulation of different and important cellular processes (Clark et al., 2014), as shown in a mouse model of ischemic brain injury, where BMSCs overexpressing miR-705 contributed to ameliorate neurological deficits and to suppress neuronal death, associated with a 2-fold increase in BDNF and VEGF expression when compared to sham and vehicle groups (Ji et al., 2017). Several studies evaluated the effects of exosomes derived from genetically modified MSCs for the treatment of neurological diseases. Exosomes derived from MSCs overexpressing pigment epithelium-derived factor (PEDF) or miR-25 showed neuroprotective effects in models of cerebral ischemia reperfusion and ischemic spinal cord by mechanisms involved in axonal preservation, such as regulation of autophagy and apoptosis or reduction of inflammation and oxidative stress (Huang X. et al., 2018; Zhao et al., 2018). MSCs overexpressing miR-21, miR-124, or miR-133b in models of intracerebral hemorrhage, SCI and stroke, respectively, increased the expression of neuronal markers, such as β-III tubulin and NF200, induced neurite remodeling and outgrowth, reduced neurological damages and promoted functional recovery after treatment (Zou et al., 2014; Xin et al., 2017; Zhang H. et al., 2018).

Transcription factors have also been expressed by MSCs for the treatment of neurological diseases. BMSCs genetically engineered to express hypoxia-inducible factor 1α (HIF-1α) led to motor functional improvement, decreased cerebral infarction and increased by 4-fold the VEGF expression, when compared to the control groups in a model of cerebral artery occlusion (Yang et al., 2014). In another study, Mash1 overexpressing-MSCs enhanced neuronal markers expression, such as NeuN and GAD67, when compared to groups treated with MSCs control cells. In addition, these cells differentiated into cells similar to neurons that showed action potential, as seen by electrophysiological characterization, demonstrating that they were functional *in vitro* (Wang K. et al., 2013).

Genetically modified MSCs have also been combined with other methodologies in order to enhance their effects, as observed in a SCI model, in which the transplantation of BMSCs overexpressing BDNF combined with platelet-rich plasma (PRP) scaffolding promoted an increase in the expression of axonal markers such as NF200, 4 and 8 weeks after transplantation, when compared to groups that received control BMSC cells. Regarding GFAP levels, the BMSC groups, when combined with PRP, showed an increase in the expression of this molecule compared to groups without PRP. In addition, a better functional recovery was observed in the BMSC-BDNF group combined with PRP, with a significant gain in the BBB index, when compared to the control groups (Zhao et al., 2013). Two studies evaluated the effects of transplanted MSCs genetically engineered to express tyrosine kinase receptor type 3 (TrkC) in combination with electroacupuncture (EA) for the treatment of SCI. An increase in the expression of NT3, an important neurotrophic factor, was seen in the spinal cord, as well as an improvement in the functional recovery in the animals which received this combined therapy. Differentiation of the MSC-TrkC into neuron-like and oligodendrocyte-like cells was also seen, with an expression of NF150 and MOSP almost twice as higher when compared to the control groups. Additionally, a reduction of degenerated myelin and an increase in the number of remyelinating and normal myelinated axons either in MSC combined with EA was observed (Ding et al., 2013, 2015).

## Clinical Trials Using Genetically Modified MSCS

To date, genetically engineered MSCs are being tested only in few clinical studies for the treatment of different diseases. Either by viral or non-viral modification, the clinical use of genetically modified MSCs raises flags about the safety of cell products and quality control that should be carefully executed to guarantee the development of safe and effective alternative treatments.

Application of modified MSCs for genetic diseases treatments is being explored in the clinical research field in cases of severe combined immunodeficiency (SCID). The standard therapy for defective adenosine deaminase (ADA) derived SCID is bone marrow transplant. However, this procedure involves high risks and compatible donors are scarce. To overcome these problems, the collection of hematopoietic stem cells and/or MSCs, derived from the patient’s bone marrow, followed by lentiviral transduction to induce correct defective ADA expression and re-implantation into the patient can be considered as a promising strategy for SCID treatment. Therefore, safety and efficiency of an improved self-inactivating lentiviral vector system for therapeutic gene delivery to patients with SCID, due to a defective ADA gene, is being studied in an interventional clinical trial (NCT03645460 ClinicalTrials.gov).

Another ongoing example of safety and efficacy evaluation of a gene transfer phase I/II clinical trial is for treating Fanconi anemia, a rare, inherited disease that is caused by a gene defect that primarily affects an individual’s bone marrow, resulting in decreased production of blood cells. In this trial, autologous bone marrow derived MSCs and hematopoietic stem cells are being transduced with a self-inactivating lentiviral vector to functionally correct the defective gene FANCA. Furthermore, the effects of an infusion of these modified cells in order to promote immune reconstitution and long-term correction of Fanconi anemia associated disease symptoms is being evaluated (NCT03351868 ClinicalTrials.gov).

In the oncology field, a first clinical trial in solid tumors was conducted for treatment of advanced gastrointestinal cancer. Subjects were treated with a combination of genetically modified autologous bone marrow derived MSCs expressing the herpes simplex virus thymidine kinase (HSV-TK) (MSC_apceth_101) and ganciclovir, in order to generate a toxic metabolite for the tumor cells. In this study, from the 10 patients treated with this genetically modified MSC, five of them reached a stable disease and a post-study observation demonstrated a median overall survival of 15.6 months. In relation to the immunological markers, a slight increase in baseline levels of IL-6 was seen in 4 of the patients, while 3 demonstrated a slight enhancement in the levels of this cytokine at the end of the study, when compared to the baseline. Additionally, a moderate improvement in baseline levels of IL-8 was seen in 5 patients and 3 of them presented an increase during the time.

Also an enhancement in TNFα/IL-10 ratio, which suggests a higher inflammatory effectiveness and an anti-tumor capacity, was observed in 5 patients as well (von Einem et al., 2019).

In addition, new anticancer immunotherapies are being developed based on the use of recombinant type I IFNs, type I IFN-encoding vectors and type I IFN-expressing cells (Zitvogel et al., 2015). Based on that, a phase I study evaluating the effects of MSCs secreting IFN-β for ovarian cancer therapy is ongoing. The study aims to determine the highest tolerable dose of human MSCs-IFN-β that can be given to patients with ovarian cancer therapy (NCT02530047 ClinicalTrials.gov). The safety and antitumor activity of a modified human MSCs are also being studied in a phase I/II study investigating the outcomes of TRAIL-overexpressing MSCs in addition to chemotherapy, in metastatic non-small cell lung cancer (NCT03298763 ClinicalTrials.gov). In another study, the maximum tolerable dose, safety and efficacy of an intratumoral injection of the IL-12-expressing Human Mesenchymal Stem Cell Vaccine GX-051 are being investigated in subjects with advanced head and neck cancer. IL-12 is a cytokine that induces the production of IFN-γ, an important anti-tumoral factor. This study will evaluate the anti-tumor response, as well as possible changes of IFN-γ and IL-12 levels in blood comparing to the baseline and changes of immune cell distribution in tumor tissue after GX-051 intratumoral injection (NCT02079324 ClinicalTrials.gov).

## Conclusion

The development of genetic engineering technologies and the continuous improvement in the knowledge of MSCs gene expression enabled the use of genetically modified MSCs with enhanced therapeutic properties compared to wild-type MSCs in a variety of disease models, as discussed in this review. Moreover, many studies described here showed that genetic modification of MSCs improved their paracrine effects, protecting viable cells in a lesion area from further damage and promoting tissue regeneration and significant functional and behavioral recovery in many animal models. However, due to issues concerning the safety and efficacy regarding the use of genetically modified MSCs for treatments, there is a gap between the experimental models and clinical trials, and the few clinical studies were conducted or are being initiated, mainly in the hematology and oncology fields. Additional preclinical studies are needed to ensure the safety and demonstrate the therapeutic potential of engineered MSCs in more relevant animal models, especially those using medium or large animal models, giving support to the translation of this therapeutic modality into a broad variety of clinical settings.

## Author Contributions

PD, TS, GS, IO, DS, JA, GGo, GGr, and MP participated in the literature search, wrote the manuscript parts, and prepared the figures and tables. RS, MD, and MS conceived the manuscript concept, wrote, and final edited the manuscript. All authors read and approved the final manuscript.

## Conflict of Interest

MD and GGr hold patents in the field of cell and gene therapy and declare a consultancy role, research funding, and stock ownership with Rigenerand Srl. The remaining authors declare that the research was conducted in the absence of any commercial or financial relationships that could be construed as a potential conflict of interest.

## References

[B1] Abbaszadeh H. A. Tiraihi T. Noori-Zadeh A. Delshad A. R. Sadeghizade M. Taheri T. (2015). Human ciliary neurotrophic factor–overexpressing stable bone marrow stromal cells in the treatment of a rat model of traumatic spinal cord injury. *Cytotherapy* 17 912–921. 10.1016/j.jcyt.2015.03.689 25939801

[B2] Allahdadi K. J. de Santana T. A. Santos G. C. Azevedo C. M. Mota R. A. Nonaka C. K. (2019). IGF-1 overexpression improves mesenchymal stem cell survival and promotes neurological recovery after spinal cord injury. *Stem Cell Res. Ther.* 10:146. 10.1186/s13287-019-1223-z 31113444 PMC6530133

[B3] Baraniak P. Mcdevitt T. (2010). Stem cell paracrine actions and tissue regeneration. *Regen. Med.* 5 121–143. 10.2217/rme.09.74 20017699 PMC2833273

[B4] Biancone L. Bruno S. Deregibus M. Tetta C. Camussi G. (2012). Therapeutic potential of mesenchymal stem cell-derived microvesicles. *Nephrol. Dial. Transpl.* 27 3037–3042. 10.1093/ndt/gfs168 22851627

[B5] Bobis-Wozowicz S. Miekus K. Wybieralska E. Jarocha D. Zawisz A. Madeja Z. (2011). Genetically modified adipose tissue-derived mesenchymal stem cells overexpressing CXCR4 display increased motility, invasiveness, and homing to bone marrow of NOD/SCID mice. *Exp. Hematol.* 39 686–696. 10.1016/j.exphem.2011.03.004 21426925

[B6] Bonilla-Porras A. R. Arevalo-Arbelaez A. Alzate-Restrepo J. F. Velez-Pardo C. Jimenez-Del-Rio M. (2018). PARKIN overexpression in human mesenchymal stromal cells from Wharton’s jelly suppresses 6-hydroxydopamine–induced apoptosis: Potential therapeutic strategy in Parkinson’s disease. *Cytotherapy* 20 45–61. 10.1016/j.jcyt.2017.09.011 29079356

[B7] Cai J. Jiao X. Zhao S. Liang Y. Ning Y. Shi Y. (2019). Transforming growth factor-β1-overexpressing mesenchymal stromal cells induced local tolerance in rat renal ischemia/reperfusion injury. *Cytotherapy* 21 535–545. 10.1016/j.jcyt.2018.12.003 30685215

[B8] Cai S. X. Liu A. R. Chen S. He H. L. Chen Q. H. Xu J. Y. (2015). Activation of Wnt/β-catenin signalling promotes mesenchymal stem cells to repair injured alveolar epithelium induced by lipopolysaccharide in mice. *Stem Cell Res. Ther.* 6:65. 10.1186/s13287-015-0060-y 25889393 PMC4414385

[B9] Cai S. X. Liu A. R. Chen S. He H. L. Chen Q. H. Xu J. Y. (2016). The orphan receptor tyrosine kinase ROR2 facilitates MSCs to repair lung injury in ARDS animal model. *Cell Transplant.* 25 1561–1574. 10.3727/096368915X689776 26531175

[B10] Campeau P. M. Rafei M. François M. Birman E. Forner K. A. Galipeau J. (2009). Mesenchymal stromal cells engineered to express erythropoietin induce anti-erythropoietin antibodies and anemia in allorecipients. *Mol. Ther.* 17 369–372. 10.1038/mt.2008.270 19088705 PMC2835073

[B11] Cantinieaux D. Quertainmont R. Blacher S. Rossi L. Wanet T. Noël A. (2013). Conditioned medium from bone marrow-derived mesenchymal stem cells improves recovery after spinal cord injury in rats: an original strategy to avoid Cell Transplantation. *PLoS One* 8:e69515. 10.1371/journal.pone.0069515 24013448 PMC3754952

[B12] Cao Y. Lu W. Sun R. Jin X. Chen L. He X. (2019). Anti-CD19 chimeric antigen receptor T cells in combination with nivolumab are safe and effective against relapsed/refractory B-cell non-hodgkin lymphoma. *Front. Oncol.* 9:767. 10.3389/fonc.2019.00767 31482064 PMC6709654

[B13] Chae Y. J. Jun D. W. Lee J. S. Saeed W. K. Kang H. T. Jang K. (2019). The use of Foxa2-overexpressing adipose tissue-derived stem cells in a scaffold system attenuates acute liver injury. *Gut Liver* 13 450–460. 10.5009/gnl18235 30602218 PMC6622567

[B14] Chen B. Chen X. Liu C. Li J. Liu F. Huang Y. (2018). Co-expression of Akt1 and Wnt11 promotes the proliferation and cardiac differentiation of mesenchymal stem cells and attenuates hypoxia/reoxygenation-induced cardiomyocyte apoptosis. *Biomed. Pharmacother.* 108 508–514. 30243083 10.1016/j.biopha.2018.09.047

[B15] Chen H.-L. Li J.-J. Jiang F. Shi W.-J. Chang G.-Y. (2020). MicroRNA-4461 derived from bone marrow mesenchymal stem cell exosomes inhibits tumorigenesis by downregulating COPB2 expression in colorectal cancer. *Biosci. Biotechnol. Biochem.* 84 338–346. 10.1080/09168451.2019.1677452 31631786

[B16] Chen L. Zhang Y. Tao L. Yang Z. Wang L. (2017). Mesenchymal stem cells with eNOS over-expression enhance cardiac repair in rats with myocardial infarction. *Cardiovasc. Drugs. Ther.* 31 9–18. 10.1007/s10557-016-6704-z 27913896

[B17] Chen X. Wu S. Tang L. Ma L. Wang F. Feng H. (2018). Mesenchymal stem cells overexpressing heme oxygenase-1 ameliorate lipopolysaccharide-induced acute lung injury in rats. *J. Cell. Physiol.* 234 7301–7319. 30362554 10.1002/jcp.27488

[B18] Chen Y. Zhao Y. Chen W. Xie L. Zhao Z. A. Yang J. (2017). MicroRNA-133 overexpression promotes the therapeutic efficacy of mesenchymal stem cells on acute myocardial infarction. *Stem Cell Res. Ther.* 8:268. 10.1186/s13287-017-0722-z 29178928 PMC5702098

[B19] Chen Y. Zuo J. Chen W. Yang Z. Zhang Y. Hua F. (2019). The enhanced effect and underlying mechanisms of mesenchymal stem cells with IL-33 overexpression on myocardial infarction. *Stem Cell Res. Ther.* 10 1–14. 10.1186/s13287-019-1392-1399 31547872 PMC6757387

[B20] Cheng Y. Gu W. Zhang G. Li X. Guo X. (2017). Activation of Notch1 signaling alleviates dysfunction of bone marrow-derived mesenchymal stem cells induced by cigarette smoke extract. *Int. J. Chron. Obstruct. Pulmon. Dis.* 12 3133–3147. 10.2147/COPD.S146201 29138545 PMC5667796

[B21] Cheng Z. Ou L. Zhou X. Li F. Jia X. Zhang Y. (2008). Targeted migration of mesenchymal stem cells modified with CXCR4 gene to infarcted myocardium improves cardiac performance. *Mol. Ther.* 16 571–579. 10.1038/sj.mt.6300374 18253156

[B22] Cho J. Zhai P. Maejima Y. Sadoshima J. (2011). Myocardial injection with GSK-3β–overexpressing bone marrow–derived mesenchymal stem cells attenuates cardiac dysfunction after myocardial infarction. *Circ. Res.* 108 478–489. 10.1161/CIRCRESAHA.110.229658 21233455 PMC3109296

[B23] Cho J. W. Lee C. Y. Ko Y. (2012). Therapeutic potential of mesenchymal stem cells overexpressing human forkhead box A2 gene in the regeneration of damaged liver tissues. *J. Gastroenterol. Hepatol.* 27 1362–1370. 10.1111/j.1440-1746.2012.07137.x 22432472 PMC3492917

[B24] Cho Y. H. Cha M. J. Song B. W. Kim I. K. Song H. Chang W. (2012). Enhancement of MSC adhesion and therapeutic efficiency in ischemic heart using lentivirus delivery with periostin. *Biomaterials* 33 1376–1385. 10.1016/j.biomaterials.2011.10.078 22112759

[B25] Choi J. S. Jeong I. S. Han J. H. Cheon S. H. Kim S. W. (2019). IL-10-secreting human MSCs generated by TALEN gene editing ameliorate liver fibrosis through enhanced anti-fibrotic activity. *Biomater. Sci.* 7 1078–1087. 10.1039/C8BM01347K 30631870

[B26] Clark E. A. Kalomoiris S. Nolta J. A. Fierro F. A. (2014). Concise review: MicroRNA function in multipotent mesenchymal stromal cells. *Stem Cells* 32 1074–1082. 10.1002/stem.1623 24860868 PMC10668871

[B27] Copland I. B. Jolicoeur E. M. Gillis M. A. Cuerquis J. Eliopoulos N. Annabi B. (2008). Coupling erythropoietin secretion to mesenchymal stromal cells enhances their regenerative properties. *Cardiovasc. Res.* 79 405–415. 10.1093/cvr/cvn090 18397963

[B28] Cui L. L. Nitzsche F. Pryazhnikov E. Tibeykina M. Tolppanen L. Rytkönen J. (2017). Integrin α4 overexpression on rat mesenchymal stem cells enhances transmigration and reduces cerebral embolism after intracarotid injection. *Stroke* 48 2895–2900. 10.1161/STROKEAHA.117.017809.) 28916665

[B29] Delcayre A. Estelles A. Sperinde J. Roulon T. Paz P. Aguilar B. (2005). Exosome display technology: applications to the development of new diagnostics and therapeutics. *Blood Cell. Mol. Dis.* 35 158–168. 10.1016/j.bcmd.2005.07.003 16087368

[B30] Deng J. Han Y. Yan C. Tian X. Tao J. Kang J. (2010). Overexpressing cellular repressor of E1A-stimulated genes protects mesenchymal stem cells against hypoxia-and serum deprivation-induced apoptosis by activation of PI3K/Akt. *Apoptosis* 15 463–473. 10.1007/s10495-009-0434-437 19997978

[B31] Ding Y. Yan Q. Ruan J. W. Zhang Y. Q. Li W. J. Zeng Z. (2013). Electroacupuncture promotes the differentiation of transplanted bone marrow mesenchymal stem cells overexpressing TrkC into neuron-like cells in transected spinal cord of rats. *Cell Transplant.* 22 65–86. 10.3727/096368912X655037 23006476

[B32] Ding Y. Zhang R. Y. He B. Liu Z. Zhang K. Ruan J. W. (2015). Combination of electroacupuncture and grafted mesenchymal stem cells overexpressing TrkC improves remyelination and function in demyelinated spinal cord of rats. *Sci. Rep.* 5 1–14. 10.1038/srep09133 25779025 PMC5390924

[B33] Di Rocco G. Gentile A. Antonini A. Truffa S. Piaggio G. Capogrossi M. C. (2012). Analysis of biodistribution and engraftment into the liver of genetically modified mesenchymal stromal cells derived from adipose tissue. *Cell Transplant.* 21 1997–2008. 10.3727/096368911X637452 22469297

[B34] Dominici M. Le Blanc K. Mueller I. Slaper-Cortenbach I. Marini F. C. Krause D. S. (2006). Minimal criteria for defining multipotent mesenchymal stromal cells. International Society for Cellular Therapy position statement. *Cytotherapy* 8 315–317. 10.1080/14653240600855905 16923606

[B35] Dong L. Li L. (2019). Large tumor suppressor gene 2-mediated Hippo signaling pathway regulates the biological behavior of mesenchymal stem cells in vitro. *Zhonghua wei zhong bing ji jiu yi xue* 31 1143–1148. 10.3760/cma.j.issn.2095-4352.2019.09.017 31657341

[B36] Donnelly J. M. Engevik A. Feng R. Xiao C. Boivin G. P. Li J. (2014). Mesenchymal stem cells induce epithelial proliferation within the inflamed stomach. *Am. J. Physiol.Gastrointest. Liver Physiol.* 306 G1075-G1088. 10.1152/ajpgi.00489.2012 24789207 PMC4059978

[B37] Du Z. Wei C. Yan J. Han B. Zhang M. Peng C. (2013). Mesenchymal stem cells overexpressing C-X-C chemokine receptor type 4 improve early liver regeneration of small-for-size liver grafts. *Liver Transpl.* 19 215–225. 10.1002/lt.23577 23193024

[B38] Duffy G. P. D’Arcy S. Ahsan T. Nerem R. M. O’Brien T. Barry F. (2010). Mesenchymal stem cells overexpressing Ephrin-B2 rapidly adopt an early endothelial phenotype with simultaneous reduction of osteogenic potential. *Tissue Eng. Part A* 16 2755–2768. 10.1089/ten.tea.2009.0623 20491587

[B39] Fierro F. A. Kalomoiris S. Sondergaard C. S. Nolta J. A. (2011). Effects on proliferation and differentiation of multipotent bone marrow stromal cells engineered to express growth factors for combined cell and gene therapy. *Stem Cells* 29 1727–1737. 10.1002/stem.720 21898687 PMC3784258

[B40] Finer M. Glorioso J. (2017). A brief account of viral vectors and their promise for gene therapy. *Gene Ther.* 24 1–2. 10.1038/gt.2016.71 28123184

[B41] Fiore E. J. Bayo J. M. Garcia M. G. Malvicini M. Lloyd R. Piccioni F. (2015). Mesenchymal stromal cells engineered to produce IGF-I by recombinant adenovirus ameliorate liver fibrosis in mice. *Stem Cell. Dev.* 24 791–801. 10.1089/scd.2014.0174 25315017 PMC4356192

[B42] Foppiani E. M. Candini O. Mastrolia I. Murgia A. Grisendi G. Samarelli A. V. (2019). Impact of HOXB7 overexpression on human adipose-derived mesenchymal progenitors. *Stem Cell Res. Ther.* 10 1–12. 10.1186/s13287-019-1200-1206 30890185 PMC6423808

[B43] Gao D. Jiang L. (2018). Exosomes in cancer therapy: a novel experimental strategy. *Am. J. Cancer Res.* 8 2165–2175. 30555736 PMC6291654

[B44] Gao X. Wu D. Dou L. Zhang H. Huang L. Zeng J. (2019). Protective effects of mesenchymal stem cells overexpressing extracellular regulating kinase 1/2 against stroke in rats. *Brain Res. Bull.* 149 42–52. 10.1016/j.brainresbull.2019.04.006 31002912

[B45] Gao X. R. Tan Y. Z. Wang H. J. (2011). Overexpression of Csx/Nkx2. 5 and GATA-4 enhances the efficacy of mesenchymal stem Cell Transplantation after myocardial infarction. *Circ. J.* 75 11,2683–2691. 10.1253/circj.CJ-11-0238 21828931

[B46] Gao Y. Yao A. Zhang W. Lu S. Yu Y. Deng L. (2010). Human mesenchymal stem cells overexpressing pigment epithelium-derived factor inhibit hepatocellular carcinoma in nude mice. *Oncogene* 29 2784–2794. 10.1038/onc.2010.38 20190814

[B47] Garcia K. D. O. Ornellas F. L. Matsumoto P. Patti C. D. L. Mello L. E. Frussa-Filho R. (2014). Therapeutic effects of the transplantation of VEGF overexpressing bone marrow mesenchymal stem cells in the hippocampus of murine model of Alzheimer’s disease. *Front Aging Neurosci.* 6:30. 10.3389/fnagi.2014.00030 24639647 PMC3945612

[B48] Gerace D. Martiniello-Wilks R. Nassif N. T. Lal S. Steptoe R. Simpson A. M. (2017). CRISPR-targeted genome editing of mesenchymal stem cell-derived therapies for type 1 diabetes: a path to clinical success? *Stem Cell Res. Ther.* 8:62 10.1186/s13287-017-0511-518 PMC5345178 28279194

[B49] Gnecchi M. Cervio E. (2013). “Mesenchymal stem cell therapy for heart disease,” in *Mesenchymal Stem Cell Therapy*, eds Chase L. G. Vemuri M. C. (Totowa, NJ: Humana Press), 241–270. 10.1007/978-1-62703-200-1_13

[B50] Gnecchi M. He H. Melo L. G. Noiseaux N. Morello F. De Boer R. A. (2009). Early beneficial effects of bone marrow-derived mesenchymal stem cells overexpressing Akt on cardiac metabolism after myocardial infarction. *Stem Cells* 27 971–979. 10.1002/stem.12 19353525 PMC2873075

[B51] Gnecchi M. He H. Noiseux N. Liang O. D. Zhang L. Morello F. (2006). Evidence supporting paracrine hypothesis for Akt-modified mesenchymal stem cell-mediated cardiac protection and functional improvement. *FASEB J.* 20 661–669. 10.1096/fj.05-5211com 16581974

[B52] Gómez-Mauricio G. Moscoso I. Martín-Cancho M. F. Crisóstomo V. Prat-Vidal C. Báez-Díaz C. (2016). Combined administration of mesenchymal stem cells overexpressing IGF-1 and HGF enhances neovascularization but moderately improves cardiac regeneration in a porcine model. *Stem Cell Res. Ther.* 7:94. 10.1186/s13287-016-0350-z 27423905 PMC4947339

[B53] Gong X. H. Liu H. Wang S. J. Liang S. W. Wang G. G. (2019). Exosomes derived from SDF1-overexpressing mesenchymal stem cells inhibit ischemic myocardial cell apoptosis and promote cardiac endothelial microvascular regeneration in mice with myocardial infarction. *J. Cell. Physiol.* 234 13878–13893. 10.1002/jcp.28070 30720220 PMC13483023

[B54] Guo Y. He J. Wu J. Yang L. Dai S. Tan X. (2008). Locally overexpressing hepatocyte growth factor prevents post-ischemic heart failure by inhibition of apoptosis via calcineurin-mediated pathway and angiogenesis. *Arch. Med. Res.* 39 179–188. 10.1016/j.arcmed.2007.11.001 18164961

[B55] Guo Y. Su L. Li Y. Guo N. Xie L. Zhang D. (2013). The synergistic therapeutic effect of hepatocyte growth factor and granulocyte colony-stimulating factor on pulmonary hypertension in rats. *Heart Vessels* 29 520–531. 10.1007/s00380-013-0395-391 23933910

[B56] Haider H. K. Jiang S. Idris N. M. Ashraf M. (2008). IGF-1–overexpressing mesenchymal stem cells accelerate bone marrow stem cell mobilization via paracrine activation of SDF-1α/CXCR4 signaling to promote myocardial repair. *Circ. Res.* 103 11,1300–1308. 10.1161/CIRCRESAHA.108.186742 18948617

[B57] Halabian R. Roudkenar M. H. Jahanian-Najafabadi A. Hosseini K. M. Tehrani H. A. (2014). Co-culture of bone marrow-derived mesenchymal stem cells overexpressing lipocalin 2 with HK-2 and HEK293 cells protects the kidney cells against cisplatin-induced injury. *Cell Biol. Int.* 39 152–163. 10.1002/cbin.10344 25049146

[B58] Han S. M. Han S. H. Coh Y. R. Jang G. Ra J. C. Kang S. K. (2014). Enhanced proliferation and differentiation of Oct4-and Sox2-overexpressing human adipose tissue mesenchymal stem cells. *Exp. Mol. Med.* 46:e101. 10.1038/emm.2014.28 24946789 PMC4081551

[B59] Han S. M. Park C. W. Ahn J. O. Park S. C. Jung W. S. Seo K. W. (2015). Pro-apoptotic and growth-inhibitory effect of IFN-β-overexpressing canine adipose tissue-derived mesenchymal stem cells against melanoma cells. *Anticancer. Res.* 35 4749–4756. 26254365

[B60] He H. Liu L. Chen Q. Liu A. Cai S. Yang Y. (2015). Mesenchymal stem cells overexpressing angiotensin-converting enzyme 2 rescue lipopolysaccharide-induced lung injury. *Cell Transplant.* 24 1699–1715. 25291359 10.3727/096368914X685087

[B61] He J. G. Li H. R. Li B. B. Xie Q. L. Yan D. Wang X. J. (2019). Bone marrow mesenchymal stem cells overexpressing GATA-4 improve cardiac function following myocardial infarction. *Perfusion* 34 696–704. 10.1177/0267659119847442 31090492

[B62] Hmadcha A. Martin-Montalvo A. Gauthier B. R. Soria B. Capilla-Gonzalez V. (2020). Therapeutic potential of mesenchymal stem cells for cancer therapy. *Front. Bioeng. Biotechnol.* 8:43. 10.3389/fbioe.2020.00043 32117924 PMC7013101

[B63] Hnatiuk A. P. Ong S. G. Olea F. D. Locatelli P. Riegler J. Lee W. H. (2016). allogeneic mesenchymal stromal cells overexpressing mutant human hypoxia-inducible factor 1-α (HIF 1-α) in an ovine model of acute myocardial infarction. *J. Am. Heart Assoc.* 5:e003714. 10.1161/JAHA.116.003714 27385426 PMC5015403

[B64] Hu J. Yang Z. Wang J. Tang Y. Liu H. Zhang B. (2013). Infusion of Trx-1-overexpressing hucMSC prolongs the survival of acutely irradiated NOD/SCID mice by decreasing excessive inflammatory injury. *PLoS One* 8:e78227. 10.1371/journal.pone.0078227 24223778 PMC3817237

[B65] Hu Q. Sun W. Wang J. Ruan H. Zhang X. Ye Y. (2018). Conjugation of haematopoietic stem cells and platelets decorated with anti-PD-1 antibodies augments anti-leukaemia efficacy. *Nat. Biomed. Eng.* 2 831–840. 10.1038/s41551-018-0310-312 31015615 PMC7032014

[B66] Hu X. Chen P. Wu Y. Wang K. Xu Y. Chen H. (2016). MiR-211/STAT5A signaling modulates migration of mesenchymal stem cells to improve its therapeutic efficacy. *Stem Cells* 34 1846–1858. 10.1002/stem.2391 27145179 PMC5096301

[B67] Huang B. Tabata Y. Gao J. Q. (2012). Mesenchymal stem cells as therapeutic agents and potential targeted gene delivery vehicle for brain diseases. *J. Control. Release* 162 464–473. 10.1016/j.jconrel.2012.07.034 22885026

[B68] Huang F. Li M. L. Fang Z. F. Hu X. Q. Liu Q. M. Liu Z. J. (2013a). Overexpression of MicroRNA-1 improves the efficacy of mesenchymal stem Cell Transplantation after myocardial infarction. *Cardiology* 125 18–30. 10.1159/000347081 23615185

[B69] Huang F. Zhu X. Hu X. Q. Fang Z. F. Tang L. Lu X. L. (2013b). Mesenchymal stem cells modified with miR-126 release angiogenic factors and activate Notch ligand Delta-like-4, enhancing ischemic angiogenesis and cell survival. *Int. J. Mol. Med.* 31 484–492. 10.3892/ijmm.2012.1200 23229021

[B70] Huang J. Zhang Z. Guo J. Ni A. Deb A. Zhang L. (2010). Genetic modification of mesenchymal stem cells overexpressing CCR1 increases cell viability, migration, engraftment, and capillary density in the injured myocardium. *Circ. Res.* 106 1753–1762. 10.1161/CIRCRESAHA.109.196030 20378860 PMC2884066

[B71] Huang S. D. Lu F. L. Xu X. Y. Liu X. H. Zhao X. X. Zhao B. Z. (2006). Transplantation of angiogenin-overexpressing mesenchymal stem cells synergistically augments cardiac function in a porcine model of chronic ischemia. *J. Thorac. Cardiovasc. Surg.* 132 1329–1338. 10.1016/j.jtcvs.2006.08.021 17140951

[B72] Huang W. Wang T. Zhang D. Zhao T. Dai B. Ashraf A. (2012). Mesenchymal stem cells overexpressing CXCR4 attenuate remodeling of postmyocardial infarction by releasing matrix metalloproteinase-9. *Stem Cell. Dev.* 21 778–789. 10.1089/scd.2011.0126 21671800 PMC3295858

[B73] Huang X. Ding J. Li Y. Liu W. Ji J. Wang H. (2018). Exosomes derived from PEDF modified adipose-derived mesenchymal stem cells ameliorate cerebral ischemia-reperfusion injury by regulation of autophagy and apoptosis. *Exp. Cell Res.* 371 269–277. 10.1016/j.yexcr.2018.08.021 30142325

[B74] Huang Y. Wang J. Cai J. Qiu Y. Zheng H. Lai X. (2018). Targeted homing of CCR2-overexpressing mesenchymal stromal cells to ischemic brain enhances post-stroke recovery partially through PRDX4-mediated blood-brain barrier preservation. *Theranostics* 8 5929–5944. 10.7150/thno.28029 30613272 PMC6299433

[B75] Huang Z. Y. Hong L. Q. Na N. Luo Y. Miao B. Chen J. (2012). Infusion of mesenchymal stem cells overexpressing GDNF ameliorates renal function in nephrotoxic serum nephritis. *Cell Biochem. and Funct.* 30 139–144. 10.1002/cbf.1827 22105543

[B76] Huleihel L. Sellares J. Cardenes N. Álvarez D. Faner R. Sakamoto K. (2017). Modified mesenchymal stem cells using miRNA transduction alter lung injury in a bleomycin model. *Am. J. Physiol. Lung Cell. Mol. Physiol.* 313 L92–L103. 10.1152/ajplung.00323.2016 28385811 PMC5538868

[B77] Jadczyk T. Tfaily E. B. Mishra S. Jêdrzejek M. Bołoz M. Padmanabhan P. (2017). *Innovative Diagnostics and Treatment: Nanorobotics and Stem Cells.* Singapore: Springer.

[B78] Jeong S. R. Kwon M. J. Lee H. G. Joe E. H. Lee J. H. Kim S. S. (2012). Hepatocyte growth factor reduces astrocytic scar formation and promotes axonal growth beyond glial scars after spinal cord injury. *Exp. Neurol.* 233 312–322. 10.1016/j.expneurol.2011.10.021 22079829

[B79] Jerkic M. Masterson C. Ormesher L. Gagnon S. Goyal S. Rabani R. (2019). Overexpression of IL-10 enhances the efficacy of human umbilical-cord-derived mesenchymal stromal cells in *E. coli Pneumosepsis*. *J. Clin. Med.* 8:847. 10.3390/jcm8060847 31200579 PMC6616885

[B80] Ji M. Wang W. Li S. Hu W. (2017). Implantation of bone mesenchymal stem cells overexpressing miRNA 705 mitigated ischemic brain injury. *Mol. Med. Rep.* 16 8323–8328. 10.3892/mmr.2017.7626 28983620

[B81] Jia Y. Wu D. Zhang R. Shuang W. Sun J. Hao H. (2014). Bone marrow-derived mesenchymal stem cells expressing the Shh transgene promotes functional recovery after spinal cord injury in rats. *Neurosci. Lett.* 573 46–51. 10.1016/j.neulet.2014.05.010 24837681

[B82] Jiang J. Wei D. Sun L. Wang Y. Wu X. Li Y. (2014). A preliminary study on the construction of double suicide gene delivery vectors by mesenchymal stem cells and the in vitro inhibitory effects on SKOV3 cells. *Oncol. Rep.* 31 781–787. 10.3892/or.2013.2898 24317390

[B83] Jiang S. Haider H. K. Idris N. M. Salim A. Ashraf M. (2006). Supportive interaction between cell survival signaling and angiocompetent factors enhances donor cell survival and promotes angiomyogenesis for cardiac repair. *Circ. Res.* 99 776–784. 10.1161/01.RES.0000244687.97719.4f 16960098

[B84] Jiang X. Fitch S. Wang C. Wilson C. Li J. Grant G. A. (2016). Nanoparticle engineered TRAIL-overexpressing adipose-derived stem cells target and eradicate glioblastoma via intracranial delivery. *Proc. Natl. Acad. Sci. U.S.A.* 113 13857–13862. 10.1073/pnas.1615396113 27849590 PMC5137687

[B85] Jiang Z. Hu X. Yu H. Xu Y. Wang L. Chen H. (2013). Human endometrial stem cells confer enhanced myocardial salvage and regeneration by paracrine mechanisms. *J. Cell Mol. Med.* 17 1247–1260. 10.1111/jcmm.12100 23837896 PMC3843975

[B86] Jin S. Li H. Han M. Ruan M. Liu Z. Zhang F. (2016). Mesenchymal stem cells with enhanced Bcl-2 expression promote liver recovery in a rat model of hepatic cirrhosis. *Cell Physiol. Biochem.* 40 1117–1128. 10.1159/000453166 27960154

[B87] Joyce N. Annett G. Wirthlin L. Olson S. Bauer G. Nolta J. A. (2010). Mesenchymal stem cells for the treatment of neurodegenerative disease. *Regen. Med.* 5 933–946. 10.2217/rme.10.72 21082892 PMC3017479

[B88] Kalimuthu S. Oh J. M. Gangadaran P. Zhu L. Lee H. W. Rajendran R. L. (2017). In vivo tracking of chemokine receptor CXCR4-engineered mesenchymal stem cell migration by optical molecular imaging. *Stem Cell. Int.* 2017:8085637. 10.1155/2017/8085637 28740515 PMC5505027

[B89] Kalluri R. LeBleu V. S. (2020). The biology, function, and biomedical applications of exosomes. *Science* 367:6478. 10.1126/science.aau6977 32029601 PMC7717626

[B90] Kang K. Ma R. Cai W. Huang W. Paul C. Liang J. (2015). Exosomes secreted from CXCR4 overexpressing mesenchymal stem cells promote cardioprotection via Akt signaling pathway following myocardial infarction. *Stem Cells Int.* 2015:659890. 10.1155/2015/659890 26074976 PMC4436515

[B91] Kanki-Horimoto S. Horimoto H. Mieno S. Kishida K. Watanabe F. Furuya E. (2006). Synthetic vascular prosthesis impregnated with mesenchymal stem cells overexpressing endothelial nitric oxide synthase. *Circulation* 114 I–327–I–330. 10.1161/CIRCULATIONAHA.105.001586 16820594

[B92] Kearns-Jonker M. Dai W. Gunthart M. Fuentes T. Yeh H. Y. Gerczuk P. (2012). Genetically engineered mesenchymal stem cells influence gene expression in donor cardiomyocytes and the recipient heart. *J. of Stem Cell Res. Ther.* 2012:005. 10.4172/2157-7633.s1-005 PMC3487477 23125947

[B93] Kim H. Park J. (2017). Usage of human mesenchymal stem cells in cell-based therapy: advantages and disadvantages. *Dev. Reprod.* 21 1–10. 10.12717/DR.2017.21.1.00 28484739 PMC5409204

[B94] Kim S. W. Lee D. W. Yu L. H. Zhang H. Z. Kim C. E. Kim J. M. (2012). Mesenchymal stem cells overexpressing GCP-2 improve heart function through enhanced angiogenic properties in a myocardial infarction model. *Cardiovasc. Res.* 95 495–506. 10.1093/cvr/cvs224 22886775

[B95] Kopru C. Z. Cagnan I. Akar I. Esendagli G. Korkusuz P. Gunel-Ozcan A. (2018). Dual effect of glucocorticoid-induced tumor necrosis factor–related receptor ligand carrying mesenchymal stromal cells on small cell lung cancer: a preliminary in vitro study. *Cytotherapy* 20 930–940. 10.1016/j.jcyt.2018.05.002 30180943

[B96] Kucic T. Copland I. B. Cuerquis J. Coutu D. L. Chalifour L. E. Gagnon R. F. (2008). Mesenchymal stromal cells genetically engineered to overexpress IGF-I enhance cell-based gene therapy of renal failure-induced anemia. *Am. J. Physiol. Renal Physiol.* 295 F488–F496. 10.1152/ajprenal.00044.2008 18524862

[B97] Kuro-o M. Matsumura Y. Aizawa H. Kawaguchi H. Suga T. Utsugi T. (1997). Mutation of the mouse klotho gene leads to a syndrome resembling ageing. *Nature* 390 45–51. 9363890 10.1038/36285

[B98] Labedz-Maslowska A. Lipert B. Berdecka D. Kedracka-Krok S. Jankowska U. Kamycka E. (2015). Monocyte chemoattractant protein-induced protein 1 (MCPIP1) enhances angiogenic and cardiomyogenic potential of murine bone marrow-derived mesenchymal stem cells. *PLoS One* 10:e0133746. 10.1371/journal.pone.0133746 26214508 PMC4516329

[B99] Lai L. Chen J. Wei X. Huang M. Hu X. Yang R. (2015). Transplantation of MSCs overexpressing HGF into a rat model of liver fibrosis. *Mol. Imaging Biol.* 18 43–51. 10.1007/s11307-015-0869-x 26194009

[B100] Lee E. J. Choi E. K. Kang S. K. Kim G. H. Park J. Y. Kang H. J. (2012). N-cadherin determines individual variations in the therapeutic efficacy of human umbilical cord blood-derived mesenchymal stem cells in a rat model of myocardial infarction. *Mol. Ther.* 20 155–167. 10.1038/mt.2011.202 22068423 PMC3255601

[B101] Lee S. H. Kim Y. Rhew D. Kim A. Jo K. R. Yoon Y. (2017). Effect of canine mesenchymal stromal cells overexpressing heme oxygenase-1 in spinal cord injury. *J. Vet. Sci.* 18 377–386. 10.4142/jvs.2017.18.3.377 27586469 PMC5639091

[B102] Lewis P. Hensel M. Emerman M. (1992). Human immunodeficiency virus infection of cells arrested in the cell cycle. *EMBO J.* 11 3053–3058. 1322294 10.1002/j.1460-2075.1992.tb05376.x PMC556788

[B103] Li B. Zhang Y. Li M. Zhao X. Xie H. Guo X. (2018). Genetic correction of adipose tissue-derived mesenchymal stem cells mediated by TALEN targeting the GDF5 gene. *Int. J. Mol. Med.* 41 2397–2405. 10.3892/ijmm.2018.3442 29393424

[B104] Li H. X. Zhou Y. F. Jiang B. Zhao X. Jiang T. B. Li X. (2014). GATA-4 induces changes in electrophysiological properties of rat mesenchymal stem cells. *Biochim. Biophys. Acta* 1840 2060–2069. 10.1016/j.bbagen.2014.02.020 24582939

[B105] Li J. Zheng C. Q. Li Y. Yang C. Lin H. Duan H. G. (2015). Hepatocyte growth factor gene-modified mesenchymal stem cells augment sinonasal wound healing. *Stem Cells Dev.* 24 1817–1830. 10.1089/scd.2014.0521 25835956 PMC4507357

[B106] Li L. Chen X. Wang W. E. Zeng C. (2016). How to improve the survival of transplanted mesenchymal stem cell in ischemic heart? *Stem Cells Int.* 2016:9682757. 10.1155/2016/9682757 26681958 PMC4670674

[B107] Li S. Siriwon N. Zhang X. Yang S. Jin T. He F. (2017). Enhanced cancer immunotherapy by chimeric antigen receptor–modified T cells engineered to secrete checkpoint inhibitors. *Clin. Cancer Res* 23 6982–6992. 10.1158/1078-0432.CCR-17-0867 28912137

[B108] Li S. H. Sun Z. Guo L. Han M. Wood M. F. Ghosh N. (2012). Elastin overexpression by cell-based gene therapy preserves matrix and prevents cardiac dilation. *J. Cell Mol. Med.* 16 2429–2439. 10.1111/j.1582-4934.2012.01560.x 22435995 PMC3823437

[B109] Li X. Wang Q. Ding L. Wang Y. X. Zhao Z. D. Mao N. (2019). Intercellular adhesion molecule-1 enhances the therapeutic effects of MSCs in a dextran sulfate sodium-induced colitis models by promoting MSCs homing to murine colons and spleens. *Stem Cell Res. Ther.* 10 1–11. 10.1186/s13287-019-1384-1389 31443680 PMC6708236

[B110] Liang J. Huang W. Yu X. Ashraf A. Wary K. K. Xu M. (2012). Suicide gene reveals the myocardial neovascularization role of mesenchymal stem cells overexpressing CXCR4 (MSCCXCR4). *PLoS One* 7:e46158. 10.1371/journal.pone.0046158 23029422 PMC3460871

[B111] Lim J. Y. Park S. I. Kim S. M. Jun J. A. Oh J. H. Ryu C. H. (2011). Neural differentiation of brain-derived neurotrophic factor-expressing human umbilical cord blood-derived mesenchymal stem cells in culture via TrkB-mediated ERK and β-catenin phosphorylation and following transplantation into the developing brain. *Cell Transplant.* 20 1855–1866. 10.3727/096368910X557236 21375801

[B112] Ling X. Marini F. Konopleva M. Schober W. Shi Y. Burks J. (2010). Mesenchymal stem cells overexpressing IFN-β inhibit breast cancer growth and metastases through Stat3 signaling in a syngeneic tumor model. *Cancer Microenviron.* 3 83–95. 10.1007/s12307-010-0041-48 21209776 PMC2990497

[B113] Liu B. Qian J. M. (2015). Cytoprotective role of heme oxygenase-1 in liver ischemia reperfusion injury. *Int. J. Clin. Exp. Med.* 8 19867–19873. 26884897 PMC4723742

[B114] Liu L. Chen J. X. Zhang X. W. Sun Q. Yang L. Liu A. (2018). Chemokine receptor 7 overexpression promotes mesenchymal stem cell migration and proliferation via secreting Chemokine ligand 12. *Sci. Rep.* 8 1–10. 10.1038/s41598-017-18509-18501 29317710 PMC5760632

[B115] Liu N. Patzak A. Zhang J. (2013). CXCR4-overexpressing bone marrow-derived mesenchymal stem cells improve repair of acute kidney injury. *Am. J. Physiol. Renal Physiol.* 305 F1064–F1073. 10.1152/ajprenal.00178.2013 23884141

[B116] Liu X. S. Li J. F. Wang S. S. Wang Y. T. Zhang Y. Z. Yin H. L. (2014). Human umbilical cord mesenchymal stem cells infected with adenovirus expressing HGF promote regeneration of damaged neuron cells in a Parkinson’s disease model. *Biomed. Res. Int.* 2014:909657. 10.1155/2014/909657 25276829 PMC4167956

[B117] Liu Z. Wang C. Wang X. Xu S. (2015). Therapeutic effects of transplantation of as-mir-937-expressing mesenchymal stem cells in murine model of alzheimer’s disease. *Cell Physiol. Biochem.* 37 321–330. 10.1159/000430356 26316079

[B118] Locatelli P. Olea F. D. Hnatiuk A. De Lorenzi A. Cerdá M. Giménez C. S. (2015). Mesenchymal stromal cells overexpressing vascular endothelial growth factor in ovine myocardial infarction. *Gene Ther.* 22 449–457. 10.1038/gt.2015.28 25789461

[B119] Loebinger M. R. Eddaoudi A. Davies D. Janes S. M. (2009). Mesenchymal stem cell delivery of TRAIL can eliminate metastatic cancer. *Cancer Res.* 69 4134–4142. 10.1158/0008-5472.CAN-08-4698 19435900 PMC2699841

[B120] Luo R. Lu Y. Liu J. Cheng J. Chen Y. (2019). Enhancement of the efficacy of mesenchymal stem cells in the treatment of ischemic diseases. *Biomed. Pharmacother.* 109 2022–2034. 10.1016/j.biopha.2018.11.068 30551458

[B121] Ma J. Zhao Y. Sun L. Sun X. Zhao X. Sun X. (2017). Exosomes derived from AKt-modified human umbilical cord mesenchymal stem cells improve cardiac regeneration and promote angiogenesis via activating platelet-derived growth factor D. *Stem Cells Transl. Med.* 6 51–59. 10.5966/sctm.2016-2038 28170176 PMC5442756

[B122] Madonna R. Angelucci S. Di Giuseppe F. Doria V. Giricz Z. Görbe A. (2019). Proteomic analysis of the secretome of adipose tissue-derived murine mesenchymal cells overexpressing telomerase and myocardin. *J. Mol. Cell Cardiol.* 131 171–186. 10.1016/j.yjmcc.2019.04.019 31055035

[B123] Madonna R. Rokosh G. De Caterina R. Bolli R. (2010). Hepatocyte growth factor/Met gene transfer in cardiac stem cells—potential for cardiac repair. *Basic Res. Cardiol.* 105 443–452. 10.1007/s00395-010-0102-107 20393738 PMC3652980

[B124] Maiti P. Peruzzaro S. Kolli N. Andrews M. Al-Gharaibeh A. Rossignol J. (2019). Transplantation of mesenchymal stem cells overexpressing interleukin-10 induces autophagy response and promotes neuroprotection in a rat model of TBI. *J Cell Mol Med.* 23 5211–5224. 10.1111/jcmm.14396 31162801 PMC6653779

[B125] Man A. J. Kujawski G. Burns T. S. Miller E. N. Fierro F. A. Leach J. K. (2016). Neurogenic potential of engineered mesenchymal stem cells overexpressing VEGF. *Cell. Mol. Bioeng.* 9 96–106. 27087859 10.1007/s12195-015-0425-4 PMC4830493

[B126] Mao J. Lv Z. Zhuang Y. (2014). MicroRNA-23a is involved in tumor necrosis factor-α induced apoptosis in mesenchymal stem cells and myocardial infarction. *Exp. Mol. Pathol.* 97 23–30. 10.1016/j.yexmp.2013.11.005 24269648

[B127] Mao Q. Lin C. Gao J. Liang X. Gao W. Shen L. (2014). Mesenchymal stem cells overexpressing integrin-linked kinase attenuate left ventricular remodeling and improve cardiac function after myocardial infarction. *Mol. Cell. Biochem.* 397 203–214. 10.1007/s11010-014-2188-y 25134935

[B128] Mao Q. Lin C. X. Liang X. L. Gao J. S. Xu B. (2013). Mesenchymal stem cells overexpressing integrin-linked kinase attenuate cardiac fibroblast proliferation and collagen synthesis through paracrine actions. *Mol. Med. Rep.* 7 1617–1623. 10.3892/mmr.2013.1348 23450431

[B129] Marofi F. Vahedi G. Biglari A. Esmaeilzadeh A. Athari S. S. (2017). Mesenchymal stromal/stem cells: a new era in the cell-based targeted gene therapy of cancer. *Front. Immunol.* 8:1770. 10.3389/fimmu.2017.01770 29326689 PMC5741703

[B130] Martínez-González I. Cruz M. J. Moreno R. Morell F. Munoz X. Aran J. M. (2014). Human mesenchymal stem cells resolve airway inflammation, hyperreactivity, and histopathology in a mouse model of occupational asthma. *Stem Cells Dev.* 23 2352–2363. 10.1089/scd.2013.0616 24798370 PMC4172465

[B131] Martínez-González I. Roca O. Masclans J. R. Moreno R. Salcedo M. T. Baekelandt V. (2013). Human mesenchymal stem cells overexpressing the IL-33 antagonist soluble IL-1 receptor–like–1 attenuate endotoxin-induced acute lung injury. *Am. J. Respir. Cell Mol. Biol.* 49 552–562. 10.1165/rcmb.2012-0406OC 23656573

[B132] Matsushita K. Morello F. Wu Y. Zhang L. Iwanaga S. Pratt R. E. (2010). Mesenchymal stem cells differentiate into renin-producing juxtaglomerular (JG)-like cells under the control of liver X receptor-α. *J. Biol. Chem.* 285 11974–11982. 10.1074/jbc.M109.099671 20118482 PMC2852935

[B133] Mayorga M. E. Kiedrowski M. McCallinhart P. Forudi F. Ockunzzi J. Weber K. (2017). Role of SDF-1: CXCR4 in impaired post-myocardial infarction cardiac repair in diabetes. *Stem Cells Transl. Med.* 7 115–124. 10.1002/sctm.17-0172 29119710 PMC5746149

[B134] Mei S. H. McCarter S. D. Deng Y. Parker C. H. Liles W. C. Stewart D. J. (2007). Prevention of LPS-induced acute lung injury in mice by mesenchymal stem cells overexpressing angiopoietin 1. *PLoS Med.* 4:e269. 10.1371/journal.pmed.0040269 17803352 PMC1961632

[B135] Meng X. Li J. Yu M. Yang J. Zheng M. Zhang J. (2017). Transplantation of mesenchymal stem cells overexpressing IL10 attenuates cardiac impairments in rats with myocardial infarction. *J. Cell. Physiol.* 233 587–595. 10.1002/jcp.25919 28322445

[B136] Meng X. Zheng M. Yu M. Bai W. Zuo L. Bu X. (2019). Transplantation of CRISPRa system engineered IL10-overexpressing bone marrow-derived mesenchymal stem cells for the treatment of myocardial infarction in diabetic mice. *J. Biol. Eng.* 13:49 10.1186/s13036-019-0163-166 PMC6543626 31164920

[B137] Meyer G. P. Wollert K. C. Lotz J. Steffens J. Lippolt P. Fichtner S. (2006). Intracoronary bone marrow cell transfer after myocardial infarction. *Circulation* 113 1287–1294. 10.1161/CIRCULATIONAHA.105.575118 16520413

[B138] Milone M. C. O’Doherty U. (2018). Clinical use of lentiviral vectors. *Leukemia* 32 1529–1541. 10.1038/s41375-018-0106-100 29654266 PMC6035154

[B139] Moradian H. Keshvari H. Fasehee H. Dinarvand R. Faghihi S. (2017). Combining NT3-overexpressing MSCs and PLGA microcarriers for brain tissue engineering: a potential tool for treatment of Parkinson’s disease. *Mater. Sci. Eng. C Mater. Biol. Appl.* 76 934–943. 10.1016/j.msec.2017.02.178 28482609

[B140] Moya A. Paquet J. Deschepper M. Larochette N. Oudina K. Denoeud C. (2018). Human mesenchymal stem cell failure to adapt to glucose shortage and rapidly use intracellular energy reserves through glycolysis explains poor cell survival after implantation. *Stem Cells* 36 363–376. 10.1002/stem.2763 29266629

[B141] Mueller L. P. Luetzkendorf J. Widder M. Nerger K. Caysa H. Mueller T. (2011). TRAIL-transduced multipotent mesenchymal stromal cells (TRAIL-MSC) overcome TRAIL resistance in selected CRC cell lines in vitro and in vivo. *Cancer Gene Ther.* 18 229–239. 10.1038/cgt.2010.68 21037557

[B142] Murphy M. Moncivais K. Caplan A. (2013). Mesenchymal stem cells: environmentally responsive therapeutics for regenerative medicine. *Exp. Mol. Med.* 45:e54. 10.1038/emm.2013.94 24232253 PMC3849579

[B143] Nakajima M. Nito C. Sowa K. Suda S. Nishiyama Y. Nakamura-Takahashi A. (2017). Mesenchymal stem cells overexpressing interleukin-10 promote neuroprotection in experimental acute ischemic stroke. *Mol. Ther. Methods Clin. Dev.* 6 102–111. 10.1016/j.omtm.2017.06.005 28725658 PMC5502709

[B144] Naso M. F. Tomkowicz B. Perry W. L. Strohl W. R. (2017). Adeno-associated virus (AAV) as a vector for gene therapy. *BioDrugs* 31 317–334. 10.1007/s40259-017-0234-235 28669112 PMC5548848

[B145] Nayak S. Herzog R. W. (2010). Progress and prospects: immune responses to viral vectors. *Gene Ther.* 17 295–304. 10.1038/gt.2009.148 19907498 PMC3044498

[B146] Ni J. Liu X. Yin Y. Zhang P. Xu Y. W. Liu Z. (2019). Exosomes derived from TIMP2-modified human umbilical cord mesenchymal stem cells enhance the repair effect in rat model with myocardial infarction Possibly by the Akt/Sfrp2 pathway. *Oxid. Med. Cell. Longev.* 2019;1958941. 10.1155/2019/1958941 31182988 PMC6512021

[B147] Noiseux N. Gnecchi M. Lopez-Ilasaca M. Zhang L. Solomon S. D. Deb A. (2006). Mesenchymal stem cells overexpressing Akt dramatically repair infarcted myocardium and improve cardiac function despite infrequent cellular fusion or differentiation. *Mol. Ther.* 14 840–850. 10.1016/j.ymthe.2006.05.016 16965940

[B148] Noori-Zadeh A. Mesbah-Namin S. A. Tiraihi T. Rajabibazl M. Taheri T. (2014). Non-viral human proGDNF gene delivery to rat bone marrow stromal cells under ex vivo conditions. *J. Neurol. Sci.* 339 81–86. 10.1016/j.jns.2014.01.025 24518204

[B149] Otani K. Yamahara K. Ohnishi S. Obata H. Kitamura S. Nagaya N. (2009). Nonviral delivery of siRNA into mesenchymal stem cells by a combination of ultrasound and microbubbles. *J Controll. Release* 133 146–153. 10.1016/j.jconrel.2008.09.088 18976686

[B150] Park J. S. Na K. Woo D. G. Yang H. N. Kim J. M. Kim J. H. (2010). Non-viral gene delivery of DNA polyplexed with nanoparticles transfected into human mesenchymal stem cells. *Biomaterials* 31 124–132. 10.1016/j.biomaterials.2009.09.023 19818490

[B151] Park J. S. Suryaprakash S. Lao Y. H. Leong K. W. (2015). Engineering mesenchymal stem cells for regenerative medicine and drug delivery. *Methods* 84 3–16. 10.1016/j.ymeth.2015.03.002 25770356 PMC4526354

[B152] Park Y. M. Han S. H. Seo S. K. Park K. A. Lee W. T. Lee J. E. (2015). Restorative benefits of transplanting human mesenchymal stromal cells overexpressing arginine decarboxylase genes after spinal cord injury. *Cytotherapy* 17 25–37. 10.1016/j.jcyt.2014.08.006 25442787

[B153] Payne N. L. Dantanarayana A. Sun G. Moussa L. Caine S. McDonald C. (2012). Early intervention with gene-modified mesenchymal stem cells overexpressing interleukin-4 enhances anti-inflammatory responses and functional recovery in experimental autoimmune demyelination. *Cell Adh. Migr.* 6 179–189. 10.4161/cam.20341 22568986 PMC3427232

[B154] Pittenger M. F. Martin B. J. (2004). Mesenchymal stem cells and their potential as cardiac therapeutics. *Circ. Res.* 95 9–20. 10.1161/01.res.0000135902.99383.6f 15242981

[B155] Preda M. B. Rønningen T. Burlacu A. Simionescu M. Moskaug J. O. Valen G. (2014). Remote transplantation of mesenchymal stem cells protects the heart against ischemia-reperfusion injury. *Stem Cells* 32 2123–2134. 10.1002/stem.1687 24578312

[B156] Pulukuri S. M. K. Gorantla B. Dasari V. R. Gondi C. S. Rao J. S. (2010). Epigenetic upregulation of urokinase plasminogen activator promotes the tropism of mesenchymal stem cells for tumor cells. *Mol. Cancer Res.* 8 1074–1083. 10.1158/1541-7786.MCR-09-0495 20663859 PMC2923682

[B157] Razban V. Lotfi A. S. Soleimani M. Ahmadi H. Massumi M. Khajeh S. (2012). HIF-1α overexpression induces angiogenesis in mesenchymal stem cells. *BioRes. Open Access* 1 174–183. 10.1089/biores.2012.9905 23514846 PMC3559201

[B158] Roudkenar M. H. Halabian R. Bahmani P. Roushandeh A. M. Kuwahara Y. Fukumoto M. (2011). Neutrophil gelatinase-associated lipocalin: a new antioxidant that exerts its cytoprotective effect independent on Heme Oxygenase-1. *Free Radic. Res.* 45 810–819. 10.3109/10715762.2011.581279 21545264

[B159] Roudkenar M. H. Pour Z. G. Halabian R. Roushandeh A. M. Parichehr Y. Baghian A. G. (2008). Lipocalin 2 acts as a cytoprotective factor against cisplatin toxicity, an in vitro study. *Daru J. Pharm. Sci.* 16 106–111.

[B160] Ryu S. Lee J. M. Bae C. A. Moon C. E. Cho K. O. (2019). Therapeutic efficacy of neuregulin 1-expressing human adipose-derived mesenchymal stem cells for ischemic stroke. *PLoS One* 14:e0222587. 10.1371/journal.pone.0222587 31560696 PMC6764745

[B161] Sage E. Thakrar R. Janes S. (2016). Genetically modified mesenchymal stromal cells in cancer therapy. *Cytotherapy* 18 1435–1445. 10.1016/j.jcyt.2016.09.003 27745603 PMC5082580

[B162] Sajic M. Hunt D. P. Lee W. Compston D. A. S. Schweimer J. V. Gregson N. A. (2012). Mesenchymal stem cells lack efficacy in the treatment of experimental autoimmune neuritis despite in vitro inhibition of T-cell proliferation. *PLoS One* 7:e30708. 10.1371/journal.pone.0030708 22359549 PMC3281026

[B163] Saraswati S. Guo Y. Atkinson J. Young P. P. (2015). Prolonged hypoxia induces MCT4 expression in MSCs resulting in a secretome that is deleterious to cardiovascular repair. *Stem Cells* 33 1333–1344. 10.1002/stem.1935 25537659 PMC4376598

[B164] Sauer V. Siaj R. Todorov T. Zibert A. Schmidt H. H. J. (2010). Overexpressed ATP7B protects mesenchymal stem cells from toxic copper. *Biochem. Biophys. Res. Commun.* 395 307–311. 10.1016/j.bbrc.2010.03.158 20362556

[B165] Scheper V. Schwieger J. Hamm A. Lenarz T. Hoffmann A. (2019). BDNF-overexpressing human mesenchymal stem cells mediate increased neuronal protection in vitro. *J. Neurosci. Res.* 97 1414–1429. 10.1002/jnr.24488 31257632 PMC6772136

[B166] Schmitz J. Owyang A. Oldham E. Song Y. Murphy E. McClanahan T. K. (2005). IL-33, an interleukin-1-like cytokine that signals via the IL-1 receptor-related protein ST2 and induces T helper type 2-associated cytokines. *Immunity* 23 479–490. 10.1016/j.immuni.2005.09.015 16286016

[B167] Seo K. W. Sohn S. Y. Bhang D. H. Nam M. J. Lee H. W. Youn H. Y. (2014). Therapeutic effects of hepatocyte growth factor-overexpressing human umbilical cord blood-derived mesenchymal stem cells on liver fibrosis in rats. *Cell Biol. Int.* 38 106–116. 10.1002/cbin.10186 24115681

[B168] Shahror R. A. Linares G. R. Wang Y. Hsueh S. C. Wu C. C. Chuang D. M. (2019). Transplantation of mesenchymal stem cells overexpressing Fibroblast Growth Factor 21 facilitates cognitive recovery and enhances neurogenesis in a mouse model of traumatic brain injury. *J. Neurotrauma* 37 14–26. 10.1089/neu.2019.6422 31298621 PMC6921331

[B169] Shao Y. Shen J. Zhou F. He D. (2018). Mesenchymal stem cells overexpressing Ang1 attenuates phosgene-induced acute lung injury in rats. *Inhal. Toxicol.* 30 313–320. 10.1080/08958378.2018.1521483 30395743

[B170] Shao Y. Zhou F. He D. Zhang L. Shen J. (2019). Overexpression of CXCR7 promotes mesenchymal stem cells to repair phosgene-induced acute lung injury in rats. *Biomed. Pharmacother.* 109 1233–1239. 10.1016/j.biopha.2018.10.108 30551373

[B171] Shen H. Cui G. Li Y. Ye W. Sun Y. Zhang Z. (2019). Follistatin-like 1 protects mesenchymal stem cells from hypoxic damage and enhances their therapeutic efficacy in a mouse myocardial infarction model. *Stem Cell Res. Ther.* 10 1–14. 10.1186/s13287-018-1111-y 30635025 PMC6330478

[B172] Shen Z. Wang J. Huang Q. Shi Y. Wei Z. Zhang X. (2018). Genetic modification to induce CXCR2 overexpression in mesenchymal stem cells enhances treatment benefits in radiation-induced oral mucositis. *Cell Death Dis.* 9 1–14. 10.1038/s41419-018-0310-x 29445104 PMC5833705

[B173] Shi B. Long X. Zhao R. Liu Z. Wang D. Xu G. (2014). Transplantation of mesenchymal stem cells carrying the human receptor activity-modifying protein 1 gene improves cardiac function and inhibits neointimal proliferation in the carotid angioplasty and myocardial infarction rabbit model. *Exp. Biol. Med.* 239 356–365. 10.1177/1535370213517619 24477823

[B174] Shi R. Z. Li Q. P. (2008). Improving outcome of transplanted mesenchymal stem cells for ischemic heart disease. *Biochem. Biophys. Res. Commun.* 376 247–250. 10.1016/j.bbrc.2008.09.004 18789897

[B175] Shimizu T. Ishikawa T. Sugihara E. Kuninaka S. Miyamoto T. Mabuchi Y. (2010). c-MYC overexpression with loss of Ink4a/Arf transforms bone marrow stromal cells into osteosarcoma accompanied by loss of adipogenesis. *Oncogene* 29 5687–5699. 10.1038/onc.2010.312 20676132

[B176] Silva D. N. Souza B. S. Azevedo C. M. Vasconcelos J. F. de Jesus P. G. Feitoza M. S. (2018a). IGF-1-Overexpressing mesenchymal stem/stromal cells promote immunomodulatory and proregenerative effects in chronic experimental chagas disease. *Stem Cells Int.* 2018:9108681. 10.1155/2018/9108681 30140292 PMC6081563

[B177] Silva D. N. Souza B. S. Vasconcelos J. F. Azevedo C. M. Valim C. X. Paredes B. D. (2018b). Granulocyte-Colony stimulating factor-overexpressing mesenchymal stem cells exhibit enhanced immunomodulatory actions through the recruitment of suppressor cells in experimental chagas disease cardiomyopathy. *Front. Immunol.* 9:1449. 10.3389/fimmu.2018.01449 30013550 PMC6036245

[B178] Silva L. H. Antunes M. A. Dos Santos C. C. Weiss D. J. Cruz F. F. Rocco P. R. (2018c). Strategies to improve the therapeutic effects of mesenchymal stromal cells in respiratory diseases. *Stem Cell Res Ther.* 9:45 10.1186/s13287-018-0802-808 PMC5828113 29482654

[B179] Somia N. Verma I. M. (2000). Gene therapy: trials and tribulations. *Nat. Rev. Genet.* 1 91–99. 10.1038/35038533 11253666

[B180] Song Y. S. Lee H. J. Doo S. H. Lee S. J. Lim I. Chang K. T. (2012). Mesenchymal stem cells overexpressing hepatocyte growth factor (HGF) inhibit collagen deposit and improve bladder function in rat model of bladder outlet obstruction. *Cell Transplant.* 21 1641–1650. 10.3727/096368912X637488 22506988

[B181] Spano C. Grisendi G. Golinelli G. Rossignoli F. Prapa M. Bestagno M. (2019). Soluble TRAIL armed human MSC as gene therapy for pancreatic cancer. *Sci. Rep.* 9 1–14. 10.1038/s41598-018-37433-37436 30742129 PMC6370785

[B182] Stewart A. N. Kendziorski G. Deak Z. M. Brown D. J. Fini M. N. Copely K. L. (2017). Co-transplantation of mesenchymal and neural stem cells and overexpressing stromal-derived factor-1 for treating spinal cord injury. *Brain Res.* 1672 91–105. 10.1016/j.brainres.2017.07.005 28734802

[B183] Tomita S. Li R. K. Weisel R. D. Mickle D. A. Kim E. J. Sakai T. (1999). Autologous transplantation of bone marrow cells improves damaged heart function. *Circulation* 100(Suppl._2), II247–II256. 10567312 10.1161/01.cir.100.suppl_2.ii-247

[B184] Torres R. Martin M. C. Garcia A. Cigudosa J. C. Ramirez J. C. Rodriguez-Perales S. (2014). Engineering human tumour-associated chromosomal translocations with the RNA-guided CRISPR–Cas9 system. *Nat. Commun.* 5 1–8. 10.1038/ncomms4964 24888982

[B185] Tyciakova S. Matuskova M. Bohovic R. Kucerova L. (2017). Mesenchymal stromal cells producing TNFα lack inhibitory effect against A375 experimental lung metastases. *Neoplasma* 64 222–227. 10.4149/neo_2017_208 28043149

[B186] Uchimura E. Yamada S. Uebersax L. Fujita S. Miyake M. Miyake J. (2007). Method for reverse transfection using gold colloid as a nano-scaffold. *J. Biosci. Bioeng.* 103 101–103. 10.1263/jbb.103.101 17298909

[B187] Unzek S. Zhang M. Mal N. Mills W. R. Laurita K. R. Penn M. S. (2007). SDF-1 recruits cardiac stem cell-like cells that depolarize in vivo. *Cell Transplant.* 16 879–886. 10.3727/096368907783338271 18293886

[B188] van den Akker G. van Beuningen H. Davidson E. B. van der Kraan P. (2016). CRISPR/CAS9 mediated genome engineering of human mesenchymal stem cells. *Osteoarthritis Cartilage* 24:S231 10.1016/j.joca.2016.01.445

[B189] Van Velthoven C. T. Sheldon R. A. Kavelaars A. Derugin N. Vexler Z. S. Willemen H. L. (2013). Mesenchymal stem Cell Transplantation attenuates brain injury after neonatal stroke. *Stroke* 44 1426–1432. 10.1161/STROKEAHA.111.000326 23539530 PMC3694433

[B190] Vargas J. E. Chicaybam L. Stein R. T. Tanuri A. Delgado-Cañedo A. Bonamino M. H. (2016). Retroviral vectors and transposons for stable gene therapy: advances, current challenges and perspectives. *J. Transl. Med.* 14 1–15. 10.1186/s12967-016-1047-x 27729044 PMC5059932

[B191] Varshney R. Ali Q. Wu C. Sun Z. (2016). Monocrotaline-induced pulmonary hypertension involves downregulation of antiaging protein klotho and eNOS activity. *Hypertension* 68 1255–1263. 10.1161/HYPERTENSIONAHA.116.08184 27672025 PMC5063703

[B192] Vemula S. V. Mittal S. K. (2010). Production of adenovirus vectors and their use as a delivery system for influenza vaccines. *Expert Opin. Biol. Ther.* 10 1469–1487. 10.1517/14712598.2010.519332 20822477 PMC2951029

[B193] von Einem J. C. Guenther C. Volk H. D. Grütz G. Hirsch D. Salat C. (2019). Treatment of advanced gastrointestinal cancer with genetically modified autologous mesenchymal stem cells: Results from the phase 1/2 TREAT-ME-1 trial. *Int. J. Cancer* 145 1538–1546. 10.1002/ijc.32230 30801698

[B194] Wang B. Wu Z. H. Lou P. Y. Chai C. Han S. Y. Ning J. F. (2019). Human bone marrow-derived mesenchymal stem cell-secreted exosomes overexpressing microRNA-34a ameliorate glioblastoma development via down-regulating MYCN. *Cell. Oncol.* 42 783–799. 10.1007/s13402-019-00461 PMC12994354 31332647

[B195] Wang C. Lv D. Zhang X. Ni Z. A. Sun X. Zhu C. (2018). Interleukin-10- overexpressing mesenchymal stromal cells induce a series of regulatory effects in the inflammatory system and promote the survival of endotoxin-induced acute lung injury in mice model. *DNA Cell Biol.* 37 53–61. 10.1089/dna.2017.3735 29072959

[B196] Wang J. Xu L. Chen Q. Zhang Y. Hu Y. Yan L. (2015). Bone mesenchymal stem cells overexpressing FGF4 contribute to liver regeneration in an animal model of liver cirrhosis. *Int. J. Clin. Exp. Med.* 8 12774–12782. 26550191 PMC4612876

[B197] Wang K. Long Q. Jia C. Liu Y. Yi X. Yang H. (2013). Over-expression of Mash1 improves the GABAergic differentiation of bone marrow mesenchymal stem cells in vitro. *Brain Res. Bull.* 99 84–94. 10.1016/j.brainresbull.2013.10.005 24144723

[B198] Wang Q. Zhang Z. Ding T. Chen Z. Zhang T. (2013). Mesenchymal stem cells overexpressing PEDF decrease the angiogenesis of gliomas. *Biosci. Rep.* 33:e00019. 10.1042/BSR20110124 22917444 PMC3561916

[B199] Wang W. Zhang Y. Yang C. Wang Y. Shen J. Shi M. (2019). Transplantation of neuregulin 4-overexpressing adipose-derived mesenchymal stem cells ameliorates insulin resistance by attenuating hepatic steatosis. *Exp. Biol. Med.* 244 565–578. 10.1177/1535370219839643 30935234 PMC6545697

[B200] Wang X. Hu Q. Mansoor A. Lee J. Wang Z. Lee T. (2006). Bioenergetic and functional consequences of stem cell-based VEGF delivery in pressure-overloaded swine hearts. *Am. J. Physiol. Heart Circ. Physiol.* 290 H1393–H1405. 10.1152/ajpheart.00871.2005 16387794

[B201] Wang Y. Li Y. Song L. Li Y. Jiang S. Zhang S. (2016). The transplantation of Akt-overexpressing amniotic fluid-derived mesenchymal stem cells protects the heart against ischemia-reperfusion injury in rabbits. *Mol. Med. Rep.* 14 234–242. 10.3892/mmr.2016.5212 27151366 PMC4918560

[B202] Wang Z. Li S. Wang Y. Zhang X. Chen L. Sun D. (2019). GDNF enhances the anti-inflammatory effect of human adipose-derived mesenchymal stem cell-based therapy in renal interstitial fibrosis. *Stem Cell Res.* 41:101605. 10.1016/j.scr.2019.101605 31706095

[B203] Wei Z. Qiao S. Zhao J. Liu Y. Li Q. Wei Z. (2019). miRNA-181a over-expression in mesenchymal stem cell-derived exosomes influenced inflammatory response after myocardial ischemia-reperfusion injury. *Life Sci.* 232:116632. 10.1016/j.lfs.2019.116632 31278944

[B204] WenBo W. Fei Z. YiHeng D. Wei W. TingMang Y. WenHao Z. (2017). Human umbilical cord mesenchymal stem cells overexpressing nerve growth factor ameliorate diabetic cystopathy in rats. *Neurochem. Res.* 42 3537–3547. 10.1007/s11064-017-2401-y 28952006

[B205] WHO (2020). *Cancer.* Available online at: https://www.who.int/health-topics/ cancer#tab=tab_1 (accessed February 18, 2019).

[B206] Wu N. Zhang Y. L. Wang H. T. Li D. W. Dai H. J. Zhang Q. Q. (2016). Overexpression of hepatocyte nuclear factor 4α in human mesenchymal stem cells suppresses hepatocellular carcinoma development through Wnt/β-catenin signaling pathway downregulation. *Cancer Biol. Ther.* 17 558–565. 10.1080/15384047.2016.1177675 27124543 PMC4910927

[B207] Wu S. P. Yang Z. Li F. R. Liu X. D. Chen H. T. Su D. N. (2017). Smad7-overexpressing rat BMSCs inhibit the fibrosis of hepatic stellate cells by regulating the TGF-β1/Smad signaling pathway. *Exp. Ther. Med.* 14 2568–2576. 10.3892/etm.2017.4836 28962196 PMC5609222

[B208] Wu S. Z. Li Y. L. Huang W. Cai W. F. Liang J. Paul C. (2017). Paracrine effect of CXCR4-overexpressing mesenchymal stem cells on ischemic heart injury. *Cell Biochem. Funct.* 35 113–123. 10.1002/cbf.3254 28233339 PMC5424893

[B209] Xia P. Wang W. Bai Y. (2013). Claudin-7 suppresses the cytotoxicity of TRAIL-expressing mesenchymal stem cells in H460 human non-small cell lung cancer cells. *Apoptosis* 19 491–505. 10.1007/s10495-013-0938-z 24242915

[B210] Xiang Q. Liao Y. Chao H. Huang W. Liu J. Chen H. (2018). ISL1 overexpression enhances the survival of transplanted human mesenchymal stem cells in a murine myocardial infarction model. *Stem Cell Res. Ther.* 9:51 10.1186/s13287-018-0803-807 PMC5828309 29482621

[B211] Xie C. Du L. Y. Guo F. Li X. Cheng B. (2019). Exosomes derived from microRNA-101-3p-overexpressing human bone marrow mesenchymal stem cells suppress oral cancer cell proliferation, invasion, and migration. *Mol. Cell. Biochem.* 458 11–26. 10.1007/s11010-019-03526-3527 31165315

[B212] Xin H. Wang F. Li Y. Lu Q. E. Cheung W. L. Zhang Y. (2017). Secondary release of exosomes from astrocytes contributes to the increase in neural plasticity and improvement of functional recovery after stroke in rats treated with exosomes harvested from microRNA 133b-overexpressing multipotent mesenchymal stromal cells. *Cell Transplant.* 26 243–257. 10.3727/096368916X693031 27677799 PMC5303172

[B213] Xu X. P. Huang L. L. Hu S. L. Han J. B. He H. L. Xu J. Y. (2018). Genetic modification of mesenchymal stem cells overexpressing angiotensin II Type 2 receptor increases cell migration to injured lung in LPS-induced acute lung injury mice. *Stem Cells Transl. Med.* 7 721–730. 10.1002/sctm.17-0279 30133167 PMC6186265

[B214] Xu Y. Shen L. Li F. Yang J. Wan X. Ouyang M. (2019). microRNA-16-5p-containing exosomes derived from bone marrow-derived mesenchymal stem cells inhibit proliferation, migration, and invasion, while promoting apoptosis of colorectal cancer cells by downregulating ITGA2. *J. Cell. Physiol.* 234 21380–21394. 10.1002/jcp.28747 31102273

[B215] Yamada Y. Sakurada K. Takeda Y. Gojo S. Umezawa A. (2007). Single-cell-derived mesenchymal stem cells overexpressing Csx/Nkx2. 5 and GATA4 undergo the stochastic cardiomyogenic fate and behave like transient amplifying cells. *Exp. Cell Res.* 313 698–706. 10.1016/j.yexcr.2006.11.012 17208226

[B216] Yang C. Liu H. Liu D. (2014). Mutant hypoxia inducible factor 1α modified bone marrow mesenchymal stem cells ameliorate cerebral ischemia. *Int. J. Mol. Med.* 34 1622–1628. 10.3892/ijmm.2014.1953 25270619

[B217] Yang C. Zhou L. Gao X. Chen B. Tu J. Sun H. (2011). Neuroprotective effects of bone marrow stem cells overexpressing glial cell line-derived neurotrophic factor on rats with intracerebral hemorrhage and neurons exposed to hypoxia/reoxygenation. *Neurosurgery* 68 691–704. 21311297 10.1227/NEU.0b013e3182098a8a

[B218] Yang F. Wu R. Jiang Z. Chen J. Nan J. Zhang N. (2018). Leptin increases mitochondrial OPA1 via GSK3-mediated OMA1 ubiquitination to enhance therapeutic effects of mesenchymal stem Cell Transplantation. *Cell Death Dis.* 9 1–17. 10.1038/s41419-018-0579-579 29748581 PMC5945599

[B219] Yang J. J. Yang X. Liu Z. Q. Hu S. Y. Du Z. Y. Feng L. L. (2012). Transplantation of adipose tissue-derived stem cells overexpressing heme oxygenase-1 improves functions and remodeling of infarcted myocardium in rabbits. *Tohoku J. f Exp. Med.* 226 231–241. 10.1620/tjem.226.231 22450704

[B220] Yang K. Park H. J. Han S. Lee J. Ko E. Kim J. (2015). Recapitulation of in vivo-like paracrine signals of human mesenchymal stem cells for functional neuronal differentiation of human neural stem cells in a 3D microfluidic system. *Biomaterials* 63 177–188. 10.1016/j.biomaterials.2015.06.011 26113074

[B221] Ye Z. Lu W. Liang L. Tang M. Wang Y. Li Z. (2019). Mesenchymal stem cells overexpressing hepatocyte nuclear factor-4 alpha alleviate liver injury by modulating anti-inflammatory functions in mice. *Stem Cell Res. Ther.* 10:149 10.1186/s13287-019-1260-1267 PMC6537220 31133062

[B222] Yi X. Wei X. Lv H. An Y. Li L. Lu P. (2019). Exosomes derived from microRNA-30b-3p-overexpressing mesenchymal stem cells protect against lipopolysaccharide-induced acute lung injury by inhibiting SAA3. *Exp. Cell Res.* 383:111454. 10.1016/j.yexcr.2019.05.035 31170401

[B223] Yu B. Yang Y. Liu H. Gong M. Millard R. W. Wang Y. G. (2016). Clusterin/Akt up-regulation is critical for GATA-4 mediated cytoprotection of mesenchymal stem cells against ischemia injury. *PLoS One* 11:e0151542. 10.1371/journal.pone.0151542 26962868 PMC4786134

[B224] Yu L. Gui S. Liu Y. Qiu X. Zhang G. Zhang X. A. (2019). Exosomes derived from microRNA-199a-overexpressing mesenchymal stem cells inhibit glioma progression by down-regulating AGAP2. *Aging* 11 5300–5318. 10.18632/aging.102092 31386624 PMC6710058

[B225] Yu Y. Zhang Q. Meng Q. Zong C. Liang L. Yang X. (2016). Mesenchymal stem cells overexpressing Sirt1 inhibit prostate cancer growth by recruiting natural killer cells and macrophages. *Oncotarget* 7 71112–71122. 10.18632/oncotarget.12737 27764779 PMC5342066

[B226] Yuan Z. Kolluri K. K. Gowers K. H. Janes S. M. (2017). TRAIL delivery by MSC-derived extracellular vesicles is an effective anticancer therapy. *J. Extracell. Vesicles* 6:1265291. 10.1080/20013078.2017.1265291 28326166 PMC5328331

[B227] Zeng B. Lin G. Ren X. Zhang Y. Chen H. (2010). Over-expression of HO-1 on mesenchymal stem cells promotes angiogenesis and improves myocardial function in infarcted myocardium. *J. Biomed. Sci.* 17:80. 10.1186/1423-0127-17-80 20925964 PMC2959016

[B228] Zeng B. Ren X. Lin G. Zhu C. Chen H. Yin J. (2008). Paracrine action of HO-1-modified mesenchymal stem cells mediates cardiac protection and functional improvement. *Cell Biol. Int.* 32 1256–1264. 10.1016/j.cellbi.2008.07.010 18692581

[B229] Zeng Y. L. Zheng H. Chen Q. R. Yuan X. H. Ren J. H. Luo X. F. (2017). Bone marrow-derived mesenchymal stem cells overexpressing MiR-21 efficiently repair myocardial damage in rats. *Oncotarget.* 8 29161–29173. 10.18632/oncotarget.16254 28418864 PMC5438721

[B230] Zhang C. Zhu Y. Wang J. Hou L. Li W. An H. (2019). CXCR4-Overexpressing Umbilical Cord Mesenchymal Stem Cells Enhance Protection against Radiation-Induced Lung Injury. *Stem Cells Int.* 2019:2457082. 10.1155/2019/2457082 30867667 PMC6379846

[B231] Zhang D. Fan G. C. Zhou X. Zhao T. Pasha Z. Xu M. (2008). Over-expression of CXCR4 on mesenchymal stem cells augments myoangiogenesis in the infarcted myocardium. *J. Mol. Cell Cardiol.* 44 281–292. 10.1016/j.yjmcc.2007.11.010 18201717 PMC2601571

[B232] Zhang F. Wan X. Cao Y. Z. Sun D. Cao C. C. (2018). Klotho gene-modified BMSCs showed elevated antifibrotic effects by inhibiting the Wnt/β-catenin pathway in kidneys after acute injury. *Cell Biol. Int* 42 1670–1679. 10.1002/cbin.11068 30358003

[B233] Zhang H. Wang Y. Lv Q. Gao J. Hu L. He Z. (2018). MicroRNA-21 overexpression promotes the neuroprotective efficacy of mesenchymal stem cells for treatment of intracerebral hemorrhage. *Front. Neurol.* 9:931. 10.3389/fneur.2018.00931 30459705 PMC6233525

[B234] Zhang J. Zhou S. Zhou Y. Feng F. Wang Q. Zhu X. (2014). Hepatocyte growth factor gene-modified adipose-derived mesenchymal stem cells ameliorate radiation induced liver damage in a rat model. *PLoS One* 9:e114670. 10.1371/journal.pone.0114670 25501583 PMC4264768

[B235] Zhang J. C. Zheng G. F. Wu L. Ou Yang L. Y. Li W. X. (2014). Bone marrow mesenchymal stem cells overexpressing human basic fibroblast growth factor increase vasculogenesis in ischemic rats. *Braz. J. Med. Biol. Res.* 47 886–894. 10.1590/1414-431X20143765 25118628 PMC4181224

[B236] Zhang S. Jiang W. Ma L. Liu Y. Zhang X. Wang S. (2017). Nrf2 transfection enhances the efficacy of human amniotic mesenchymal stem cells to repair lung injury induced by lipopolysaccharide. *J. Cell. Biochem.* 119 1627–1636. 10.1002/jcb.26322 28905450

[B237] Zhang X. Chen J. Xue M. Tang Y. Xu J. Liu L. (2019). Overexpressing p130/E2F4 in mesenchymal stem cells facilitates the repair of injured alveolar epithelial cells in LPS-induced ARDS mice. *Stem Cell Res. Ther.* 10:74 10.1186/s13287-019-1169-1161 PMC6404316 30841904

[B238] Zhang Y. Li R. Rong W. Han M. Cui C. Feng Z. (2018). Therapeutic effect of hepatocyte growth factor-overexpressing bone marrow-derived mesenchymal stem cells on CCl4-induced hepatocirrhosis. *Cell Death Dis.* 9 1–12. 10.1038/s41419-018-1239-1239 30538216 PMC6290007

[B239] Zhang Z. Zhu L. Feng P. Tan Y. Zhang B. Gao E. (2019). C1q/tumor necrosis factor-related protein-3-engineered mesenchymal stromal cells attenuate cardiac impairment in mice with myocardial infarction. *Cell Death Dis.* 10 1–15. 10.1038/s41419-019-1760-1765 PMC6624206 31296837

[B240] Zhao H. Cheng L. Du X. Hou Y. Liu Y. Cui Z. (2014). Transplantation of cerebral dopamine neurotrophic factor transducted BMSCs in contusion spinal cord injury of rats: promotion of nerve regeneration by alleviating neuroinflammation. *Mol. Neurobiol.* 53 187–199. 10.1007/s12035-014-9000-9006 25421210

[B241] Zhao L. Hu C. Zhang P. Jiang H. Chen J. (2019). Preconditioning strategies for improving the survival rate and paracrine ability of mesenchymal stem cells in acute kidney injury. *J Cell Mol Med.* 23 720–730. 10.1111/jcmm.14035 30484934 PMC6349184

[B242] Zhao L. Jiang X. Shi J. Gao S. Zhu Y. Gu T. (2018). Exosomes derived from bone marrow mesenchymal stem cells overexpressing microRNA-25 protect spinal cords against transient ischemia. *J. Thorac. Cardiovasc. Surg.* 157 508–517. 10.1016/j.jtcvs.2018.07.095 30224076

[B243] Zhao L. Liu X. Zhang Y. Liang X. Ding Y. Xu Y. (2016). Enhanced cell survival and paracrine effects of mesenchymal stem cells overexpressing hepatocyte growth factor promote cardioprotection in myocardial infarction. *Exp. Cell Res.* 344 30–39. 10.1016/j.yexcr.2016.03.024 27025401

[B244] Zhao T. Yan W. Xu K. Qi Y. Dai X. Shi Z. (2013). Combined treatment with platelet-rich plasma and brain-derived neurotrophic factor-overexpressing bone marrow stromal cells supports axonal remyelination in a rat spinal cord hemi-section model. *Cytotherapy* 15 792–804. 10.1016/j.jcyt.2013.04.004 23731762

[B245] Zheng M. Duan J. He Z. Wang Z. Mu S. Zeng Z. (2017). Transplantation of bone marrow stromal stem cells overexpressing tropomyosin receptor kinase A for peripheral nerve repair. *Cytotherapy* 19 916–926. 10.1016/j.jcyt.2017.04.007 28571657

[B246] Zheng X. B. He X. W. Zhang L. J. Qin H. B. Lin X. T. Liu X. H. (2019). Bone marrow-derived CXCR4-overexpressing MSCs display increased homing to intestine and ameliorate colitis-associated tumorigenesis in mice. *Gastroenterol. Rep.* 7 127–138. 10.1093/gastro/goy017 30976426 PMC6454852

[B247] Zheng Y. B. Zhang X. H. Huang Z. L. Lin C. S. Lai J. Gu Y. R. (2012). Amniotic-fluid–derived mesenchymal stem cells overexpressing interleukin-1 receptor antagonist improve fulminant hepatic failure. *PLoS One* 7:e41392. 10.1371/journal.pone.0041392 22844472 PMC3402415

[B248] Zhou L. Lin Q. Wang P. Yao L. Leong K. Tan Z. (2017). Enhanced neuroprotective efficacy of bone marrow mesenchymal stem cells co-overexpressing BDNF and VEGF in a rat model of cardiac arrest-induced global cerebral ischemia. *Cell Death Dis.* 8:e2774. 10.1038/cddis.2017.184 28492549 PMC5520708

[B249] Zhu G. Pei L. Lin F. Yin H. Li X. He W. (2019). Exosomes from human-bone-marrow-derived mesenchymal stem cells protect against renal ischemia/reperfusion injury via transferring miR-199a-3p. *J. Cell. Physiol.* 234 23736–23749. 10.1002/jcp.28941 31180587

[B250] Zitvogel L. Galluzzi L. Kepp O. Smyth M. J. Kroemer G. (2015). Type I interferons in anticancer immunity. *Nat. Rev. Immunol.* 15 405–414. 10.1038/nri3845 26027717

[B251] Zou D. Chen Y. Han Y. Lv C. Tu G. (2014). Overexpression of microRNA-124 promotes the neuronal differentiation of bone marrow-derived mesenchymal stem cells. *Neural Regen. Res.* 9 1241–1248. 10.4103/1673-5374.135333 25206789 PMC4146284

[B252] Zuo S. Jones W. K. Li H. He Z. Pasha Z. Yang Y. (2012). Paracrine effect of Wnt11-overexpressing mesenchymal stem cells on ischemic injury. *Stem Cells Dev.* 21 598–608. 10.1089/scd 21463175 PMC3156900

